# Baicalin-Chlorogenic Acid Self-Assembled Nanoparticles: A Carrier-Free Nano-Formulation for the Treatment of Acute Pharyngitis

**DOI:** 10.3390/biomedicines14081724

**Published:** 2026-07-31

**Authors:** Xinyi Wang, Zhouyang Qian, Zhenchao Gong, Lu Sun, Liang Feng, Yanjun Yang, Xiaobin Jia

**Affiliations:** 1 Affiliated Jiangning Hospital of Chinese Medicine, School of Traditional Chinese Pharmacy, Innovation Center for Industry-Education Integration of Pediatrics and Traditional Chinese Medicine, China Pharmaceutical University, Nanjing 211198, China; 2 Nanjing Jiangning Hospital of Chinese Medicine, Nanjing 211100, China; 3 Jiangsu Key Laboratory of Chinese Medicine and Characteristic Preparations for Paediatrics, Jumpcan Pharmaceutical Co., Ltd., Taixing 225400, China

**Keywords:** acute pharyngitis, baicalin-chlorogenic acid nanoparticles, supramolecular self-assembly, TLR4/MyD88/NF-*κ*B signaling pathway, Chinese medicine delivery system

## Abstract

**Background/Objectives**: Baicalin (BA), a natural flavonoid with anti-inflammatory activity, shows promise for treating acute pharyngitis (AP) but its clinical application is hindered by poor water solubility and low oral bioavailability. Based on the clinically validated traditional Chinese medicine formula Pudilan Oral Liquid, we identified that BA and chlorogenic acid (CGA) can self-assemble into nanocomplexes (BA-CGA@NPs). This study aims to construct such a nanocomplex to enhance BA absorption and anti-AP efficacy with favorable biocompatibility. **Methods**: BA-CGA@NPs were prepared via supramolecular self-assembly and characterized by dynamic light scattering (DLS), X-ray diffraction (XRD), transmission electron microscopy (TEM), Fourier-transform infrared spectroscopy (FT-IR), differential scanning calorimetry (DSC), thermogravimetric analysis (TGA), and molecular dynamics simulation (MDS). In vivo pharmacokinetics evaluated BA absorption. Biocompatibility and therapeutic efficacy were assessed using lipopolysaccharide (LPS)-stimulated RAW264.7 macrophages and an AP rat model. **Results**: BA-CGA@NPs were successfully formed with enhanced biocompatibility. In vitro, they reduced nitric oxide (NO), reactive oxygen species (ROS), tumor necrosis factor-alpha (TNF-*α*), and interleukin-1 beta (IL-1*β*) in macrophages. In AP rats, oral BA-CGA@NPs significantly increased systemic BA exposure, ameliorated pharyngeal histopathology, and lowered IL-1*β*, TNF-*α*, and interleukin-6 (IL-6) in serum and pharyngeal tissue, outperforming free BA or CGA alone. Mechanistic studies suggested that the anti-inflammatory effect was associated with modulation of the Toll-like receptor 4 (TLR4)/MyD88/Nuclear factor-κB (NF-*κ*B) signaling pathway. **Conclusions**: Self-assembled BA-CGA@NPs enhance BA absorption and biocompatibility, alleviating AP inflammation through mechanisms associated with modulation of the TLR4/MyD88/NF-*κ*B pathway, offering a promising nano-traditional Chinese medicine strategy for poorly soluble active ingredients.

## 1. Introduction

Acute pharyngitis (AP) is an upper respiratory tract infection affecting the pharyngeal mucosa, submucosal and lymphoid tissues. It is prone to outbreaks during the spring and winter seasons [1]. Its main etiologies include bacterial or viral infections, as well as non-infectious factors such as irritating gases (e.g., ammonia), dust, smoke, tobacco use, and alcohol consumption [2,3]. Characterized by rapid onset and progression, AP can easily develop into other diseases such as pneumonia, acute edematous pharyngitis, and acute otitis media, posing serious threats to human health, daily activities, and productivity [4]. Current commonly used clinical drugs include Aspirin, non-steroidal anti-inflammatory drugs (NSAIDs), and glucocorticoids. However, these drugs are associated with limitations such as a narrow therapeutic scope, high recurrence rates, a tendency to induce drug resistance, and numerous side effects [5,6]. They exhibit poor tolerance in patients, which restricts their widespread application. Consequently, there is an urgent need to discover and develop safe and effective drugs for the treatment of AP.

**Figure 1:**
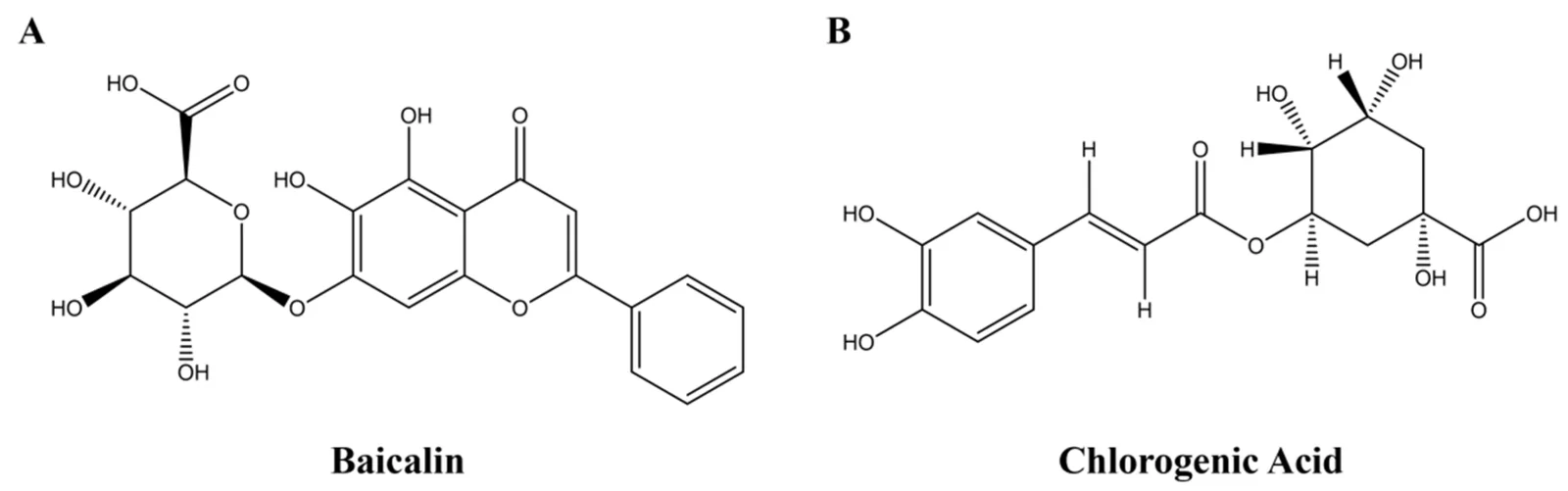
Chemical structures of (**A**) baicalin (BA) and (**B**) chlorogenic acid (CGA).

In recent years, TCM has garnered widespread attention as a research focus for potential alternative therapies due to its cost-effectiveness, high accessibility, and relatively fewer adverse reactions [7,8]. Baicalin (BA) (Figure 1A), as the primary flavonoid component of *Scutellaria baicalensis* Georgi, features straightforward extraction and low cost [9,10]. Research has confirmed its anti-inflammatory effects through the inhibition of signaling pathways such as Nuclear factor-κB (NF-*κ*B) and JAK/STAT, and the regulation of Th1/Th2 balance, demonstrating its therapeutic potential for AP [11,12,13,14]. However, the development and utilization of BA are significantly hindered by its inherent limitations, including low bioavailability, poor permeability, and poor aqueous solubility.

To overcome the aforementioned pharmaceutical limitations, nanocarrier strategies have been widely employed to enhance the delivery efficiency of poorly soluble drugs [15,16]. With the advancement of supramolecular self-assembly technology, incorporating small molecules as components of biomedical functional delivery carriers to construct drug delivery systems without any structural modification or organic solvents has become a current research focus [17,18,19,20]. Notably, BA possesses self-assembly properties and can form nanostructures through non-covalent interactions with other small-molecule compounds. For instance, assembly with berberine hydrochloride can enhance antibacterial activity [21,22,23], while combination with epigallocatechin gallate (EGCG) can significantly alleviate pulmonary inflammation and improve symptoms of acute lung injury, among other effects [24].

Based on the therapeutic concept of multi-component, multi-target combined action in TCM compound prescriptions, this study conducted an in-depth analysis of the classic and clinically proven proprietary TCM, Pudilan Oral Liquid, used for treating AP. It was discovered that chlorogenic acid (CGA) (Figure 1B) and BA, as the key pharmacodynamic components of this formula [25], can undergo self-assembly through intermolecular interactions to form a nanocomplex (BA-CGA@NPs). This assembly not only significantly improved the water solubility and permeability of BA but also demonstrated superior anti-inflammatory efficacy compared to either component alone in both RAW264.7 cell models and AP rat models, while also increasing the systemic exposure of BA. This paper systematically investigated the self-assembly behavior, formation mechanism, therapeutic effects, and underlying mechanisms of BA and CGA against AP. The aim is to provide a novel nano-TCM strategy based on the principles of TCM formula compatibility for the clinical treatment of AP, while simultaneously opening new avenues for the development and utilization of BA preparations.

## 2. Materials and Methods

### 2.1. Materials

CGA (Batch No.: PSD240227-04; purity ≥ 98%) and BA (Batch No.: PS010447; purity > 98%) were purchased from Chengdu Pusi Biotechnology Co., Ltd. (Chengdu, China). Aspirin enteric-coated tablets (National Drug Approval Number: HJ20160685, Bayer Healthcare Co., Ltd., Leverkusen, Germany) were used.

The RAW264.7 cells were purchased from KeyGEN BioTECH Co., Ltd. (Nanjing, China). The cell culture medium consisted of DMEM (Gibco, Thermo Fisher Scientific, Waltham, MA, USA, Lot No.: 6125371) supplemented with 10% fetal bovine serum (FBS, Ozfan, Guangzhou, China, Lot No.: JMSA01102) and 1% penicillin-streptomycin-gentamicin solution (Beyotime, Shanghai, China, Lot No.: A060250528). Cells in culture flasks were maintained in an incubator at 37 °C with 5% CO_2_. Subculturing, cryopreservation, and seeding for experiments were performed when cell confluency reached >90%.

Forty-eight male specific pathogen-free (SPF) Sprague-Dawley (SD) rats (250–300 g, 7–8 weeks old) were supplied by Sibeifu (Suzhou) Biotechnology Co., Ltd. (Taicang, Suzhou, Jiangsu, China) (License No.: SCXK(SU)2022-0006). All animals were acclimatized for one week prior to the experiment, with free access to food and water under controlled environmental conditions. Before the experiment, all rats were fasted for 16 h with free access to water. All animal experiments were approved by the Animal Care Committee of China Pharmaceutical University (Ethical Approval No.: 2024-08-083, Approval Date: 3 September 2024) and conducted in compliance with applicable international, national, and institutional guidelines for the care and use of laboratory animals. Procedures were carried out in strict accordance with established guidelines for animal welfare.

### 2.2. Molecular Dynamics Simulation (MDS)

The initial structures ofCGA and BA were obtained from PubChem (https://pubchem.ncbi.nlm.nih.gov/) (accessed on 7 July 2026). Deprotonated molecular models were constructed using GaussView 6.0, and all initial geometries were optimized with Gaussian16. RESP charges were calculated using Multiwfn (Version 2026.1.7) [26,27], and the General AMBER Force Field (GAFF) parameters for small molecules were generated via Sobtop [28]. GAFF is the small-molecule branch of the AMBER force field family; it was selected because BA and CGA are drug-like organic molecules, and GAFF has been extensively validated for describing the conformational behavior and intermolecular interactions of small organic compounds in aqueous environments [29,30]. All simulations were performed within the AMBER force field framework using the Gromacs-2025.2 software package.

The simulation systems were constructed with a 1:1 molar ratio of CGA to BA, by randomly placing 20 CGA and 20 BA molecules into a cubic box of 9 × 9 × 9 nm^3^. The protonation states were assigned according to the pH conditions: at pH 1.2, both molecules were modeled in their neutral forms; at pH 6.8, the carboxyl groups were fully deprotonated; at pH 8.3, the carboxyl groups and one phenolic hydroxyl group of BA were deprotonated. The box was solvated with water molecules, and counterions were added to neutralize each system. Periodic boundary conditions were applied throughout.

Each system underwent 100,000 steps of energy minimization using the steepest descent algorithm to eliminate unfavorable steric clashes. After minimization, a 200 ps NVT ensemble equilibration was performed. The non-bonded interaction cutoff distance was set to 12 Å. Long-range electrostatic interactions were treated using the particle mesh Ewald (PME) method [31]. The temperature was maintained at 298 K using the V-rescale thermostat with a coupling constant of 0.1 ps, and the pressure was stabilized at 1 bar employing the C-rescale barostat. All bonds involving hydrogen atoms were constrained using the LINCS algorithm. The integration time step was 2 fs, and a production simulation of 100 ns was carried out for each system. Post-simulation analysis was performed using the generated trajectory files, and molecular graphics were visualized with VMD [32].

### 2.3. Preparation of BA-CGA Nanoparticles (BA-CGA@NPs) and Quantitative Determination of Their Components

Based on preliminary studies [25] and MDS results, an equimolar ratio of BA to CGA was adopted, at which the apparent solubility of the poorly water-soluble BA was maximized. CGA was dissolved in pure water, followed by the addition of an equimolar amount of BA. The mixture was then ultrasonicated for 2.5 h to facilitate dissolution. Subsequently, it was centrifuged at 3000 rpm for 10 min. The supernatant was collected and lyophilized to obtain the BA-CGA@NPs. The composition of BA-CGA@NPs was determined using a Waters high-performance liquid chromatography (HPLC) system (Waters Corporation, Milford, MA, USA). Detailed experimental procedures are provided in Section S2 of the Supplementary Materials.

### 2.4. Critical Micelle Concentration (CMC) Determination

To determine the CMC of the BA-CGA polymer, pyrene was used as a fluorescent probe. Pyrene was dissolved in acetone to prepare a solution with a concentration of 12 × 10^−3^ mg/mL. The acetone was then evaporated under light-protected conditions. Different amounts of BA-CGA@NPs were added to the residue, followed by dilution to obtain solutions with final concentrations of 0.1, 0.05, 0.02, 0.01, 5 × 10^−3^, 1 × 10^−3^, and 1 × 10^−4^ mg/mL. The solutions were oscillated for 30 min and subsequently placed in a 45 °C water bath for 2 h. After cooling to 40 °C, the solutions were kept at a constant temperature overnight. The fluorescence spectra of each cooled solution were measured at room temperature using a Shimadzu RF-5301PC spectrofluorometer (Shimadzu Corporation, Kyoto, Japan), with an excitation wavelength of 335 nm and an emission range of 340–450 nm. The CMC value was determined by calculating the fluorescence intensity ratio of the first peak (I_1_, 373 nm) to the third peak (I_3_, 384 nm). The intensity ratio (I_1_/I_3_) was plotted against the logarithm of the sample concentration (log C), and the CMC was obtained from the intersection of the two linear regression lines.

### 2.5. Dynamic Light Scattering (DLS)

Appropriate quantities of CGA, BA, and BA-CGA@NPs powders were separately dissolved in ultrapure water. From each solution, 1 mL was placed into a sample cell and equilibrated at 37 °C for 1 min. Subsequently, the average particle size, polydispersity index (PDI), and Zeta potential (*ζ*, mV) of the samples were measured using a DLS instrument (Zetasizer Nano-ZS90, Malvern, UK). Each sample was measured in triplicate, and the average value was calculated.

### 2.6. Stability in Simulated Gastrointestinal Fluids

Simulated gastric fluid (SGF, pH 1.2) and simulated intestinal fluid (SIF, pH 6.8) were prepared according to the United States Pharmacopeia (USP) specifications without digestive enzymes, to avoid interference with DLS measurements. SGF was prepared by dissolving 2.0 g of NaCl in 800 mL of deionized water, adding 7.0 mL of concentrated HCl (37%), and diluting to 1 L with deionized water. SIF was prepared by dissolving 6.8 g of KH_2_PO_4_ and 0.616 g of NaOH in 800 mL of deionized water, and diluting to 1 L after pH adjustment to 6.8 ± 0.1. Both solutions were filtered through a 0.22 μm membrane prior to use.

To independently assess the behavior of BA-CGA@NPs under gastric and intestinal conditions, the freshly prepared nanocomplex was directly dispersed into each simulation solution separately. For SGF stability, BA-CGA@NPs were incubated in SGF at 37 °C, and aliquots were withdrawn at 0, 0.5, 1, and 2 h for the average particle size and polydispersity index (PDI) measurement by DLS. For SIF stability, freshly prepared BA-CGA@NPs were separately incubated in SIF at 37 °C, with sampling at 0, 1, 2, 3, 4, and 6 h.

### 2.7. Transmission Electron Microscope (TEM)

Appropriate quantities of BA, CGA, and BA-CGA@NPs powders were separately dissolved in ultrapure water. A 10 μL aliquot of each sample solution was dropped onto a copper grid and allowed to settle for 1 min. The excess liquid was then carefully removed using filter paper. Subsequently, 10 μL of phosphotungstic acid staining solution was applied onto the grid and left to settle for 1 min, after which the residual liquid was removed. The grid was dried at room temperature. Samples were imaged using TEM (Hitachi HT-7800, Hitachi High-Tech, Tokyo, Japan) operating at an acceleration voltage of 80–120 kV to observe the nanoscale morphology of the samples.

### 2.8. X-Ray Diffraction (XRD)

XRD patterns of BA, CGA, BA-CGA@NPs, and the physical mixture of BA and CGA (BA-CGA@PM) were obtained using a Bruker D8 Advance X-ray diffractometer (Bruker AXS, Karlsruhe, Germany) with Cu-K*α* radiation (λ = 1.54056 Å). The samples were analyzed directly in powder form. All measurements were performed over a 2θ range from 3° to 40°.

### 2.9. Thermogravimetric Analysis (TGA) and Differential Scanning Calorimetry (DSC)

The thermal behavior of BA, CGA, BA-CGA@NPs, and BA-CGA@PM was analyzed using a TGA (PerkinElmer TGA 4000, PerkinElmer, Llantrisant, Wales, UK) and a DSC (Netzsch DSC 3500, NETZSCH-Gerätebau, Selb, Germany). The samples were analyzed directly in powder form. The experimental conditions were as follows: a continuous nitrogen flow (purity 99.999%) and a heating rate of 10 °C/min from 25 °C to 400 °C.

### 2.10. Fourier-Transform Infrared (FT-IR) Spectroscopy

FT-IR spectra of BA, CGA, BA-CGA@NPs, and BA-CGA@PM were obtained using a Bruker Tensor 27 FT-IR spectrometer (Bruker Optik, Ettlingen, Germany). The samples were analyzed directly in powder form. The spectrophotometer was operated over a wavenumber range from 400 cm^−1^ to 4000 cm^−1^.

### 2.11. Biocompatibility

RAW264.7 cells were seeded into 96-well plates at a density of 5 × 10^3^ cells per well, with 100 μL of culture medium per well. The cells were then cultured in an incubator for 24 h. Experimental drugs were prepared using serum-free medium. Three drug solutions—BA, CGA, and BA-CGA@NPs—were prepared at concentrations of 0, 1.5625, 3.125, 6.25, 12.5, 25, 50, and 100 μg/mL. After 24 h of drug treatment, cytotoxicity was assessed using the CCK-8 assay kit (Beyotime, Cat No.: C0038). The optical density (OD) at 450 nm was measured using a microplate reader (Thermo Multiskan GO, Thermo Fisher Scientific, Waltham, MA, USA).

### 2.12. Live/Dead Cell Staining

RAW264.7 cells were seeded into 12-well plates at a density of 2 × 10^5^ cells per well, with 1 mL of culture medium per well. The cells were cultured in an incubator for 24 h. For the treatment groups (BA, CGA, and BA-CGA@NPs), the cells were cultured in serum-free medium containing 50 μg/mL of the corresponding drugs, while the blank control group received serum-free medium alone (without drugs). After 24 h of incubation, the medium was aspirated and discarded. Each well was gently rinsed once with sterile 1× PBS. Subsequently, 500 μL of Calcein AM/PI detection working solution (Beyotime, Cat No.: C2015M; prepared by mixing Calcein AM: PI: detection buffer at a volume ratio of 1:1:1000) was added to each well. The plates were protected from light and incubated for 30 min. Staining results were observed and imaged under an Axio Vert.A1 inverted fluorescence microscope (Carl Zeiss Microscopy GmbH, Jena, Germany).

### 2.13. Cell Apoptosis

Cells were cultured and treated following the same conditions described in Section 2.12. After 24 h of treatment, the medium was discarded. Each well was gently rinsed once with sterile 1× PBS. Subsequently, 195 μL of Annexin V Binding Buffer, 5 μL of Annexin V-FITC, and 10 μL of Propidium Iodide (PI) staining solution (GLPBIO Technology LLC, Montclair, CA, USA, Cat No.: GK10037) were sequentially added and mixed thoroughly. The mixture was incubated at room temperature (20–25 °C) in the dark for 10–20 min. The staining results were then examined using an Axio Vert.A1 inverted fluorescence microscope (Carl Zeiss Microscopy GmbH, Jena, Germany).

### 2.14. Scratch Test

RAW264.7 cells were seeded into 6-well plates at a density of 6 × 10^5^ cells per well, with 2 mL of culture medium per well. A reference mark was made along the edge of the plate. When cell confluency reached 90–100%, straight scratches were created using a 200 μL pipette tip, with a spacing of 0.5–1 cm between adjacent scratches. After scratching, the wells were gently rinsed once with sterile 1× PBS. For the treatment groups (BA, CGA, and BA-CGA@NPs), the cells were cultured in serum-free medium containing 50 μg/mL of the corresponding drugs, while the blank control group received serum-free medium alone (without drugs). Cell migration was observed and imaged under an inverted fluorescence microscope using brightfield illumination at 0 h, 6 h, 12 h, and 24 h after scratching.

### 2.15. NO Determination

RAW264.7 cells were seeded into 96-well plates at a density of 5 × 10^3^ cells per well, with 100 μL of culture medium per well. The cells were cultured in an incubator for 24 h. The culture medium was then discarded. Except for the control group, which received serum-free medium, all other groups were treated with 0.5 μg/mL LPS solution prepared in serum-free medium to induce inflammation. After 4 h of LPS induction, the medium in the drug treatment groups (BA, CGA, and BA-CGA@NPs) was replaced with serum-free medium containing 50 μg/mL of the corresponding drugs, while the control and model groups received fresh serum-free medium. After an additional 20 h of incubation, the cell culture supernatant was collected, and the NO content was measured using an NO assay kit (Beyotime, Cat No.: S0021S).

### 2.16. ELISA for the Determination of IL-6, TNF-α, and IL-1β Contents

The cell culture supernatant collected in Section 2.15 was used to measure the levels of IL-6, TNF-*α*, and IL-1*β* with an ELISA kit (Enzyme-linked immunosorbent assay, Cat No.: 202507). The OD at 450 nm was measured using a microplate reader. The concentrations of IL-6, TNF-*α*, and IL-1*β* in the samples were calculated based on the corresponding standard curves.

### 2.17. Content of ROS

RAW264.7 cells were seeded into 12-well plates at a density of 1 × 10^5^ cells per well, with 1 mL of culture medium per well, and cultured in an incubator for 24 h. The medium was then discarded. Except for the control group, which received serum-free medium, all other groups were treated with 0.5 μg/mL LPS solution prepared in serum-free medium. After 4 h of LPS induction, the medium in the treatment groups (BA, CGA, and BA-CGA@NPs) was replaced with serum-free medium containing 50 μg/mL of the corresponding drugs, while the control and model groups received fresh serum-free medium. Following an additional 20 h of incubation, cells were stained using the DCFH-DA fluorescent probe (Suzhou UELANDY Biotechnology Co., Ltd., Suzhou, Jiangsu, China, Cat No.: R6033). DCFH-DA was diluted 1:1000 in serum-free medium to a final concentration of 10 μM, and 500 μL of the solution was added to each well. The cells were incubated at 37 °C in the dark for 30 min. Subsequently, the cells were washed 1–2 times with serum-free medium. The staining results were examined under an inverted fluorescence microscope.

RAW264.7 cells were seeded into 96-well plates at a density of 5 × 10^3^ cells per well, with 100 μL of culture medium per well. The cells were treated following the method described above (including LPS induction and drug treatment). After staining, the fluorescence intensity was measured using a fluorescence microplate reader (Synergy Mx, BioTek, Winooski, VT, USA) at excitation/emission wavelengths of 504/529 nm.

### 2.18. In Vivo Pharmacokinetic Experiments

Male SD rats weighing 250–300 g were fasted for 12 h with free access to water, and then randomly divided into two groups (*n* = 6 per group): the BA group and the BA-CGA@NPs group. The administered dose was 200 mg/kg. The dose of 200 mg/kg was selected based on preliminary experiments showing that a lower dose resulted in plasma concentrations below the lower limit of quantification for free BA, precluding reliable pharmacokinetic modeling. This higher dose allowed accurate quantification of BA in both groups and a fair comparison of absorption profiles. Anesthesia was induced by intraperitoneal injection of 2% sodium pentobarbital (cat: P3761-5G; Sigma-Aldrich, St. Louis, MO, USA) at a dose of 40 mg/kg body weight. After oral gavage, blood samples were collected from the rats under anesthesia at predetermined time intervals (0 h, 0.25 h, 0.5 h, 0.75 h, 1 h, 2 h, 4 h, 6 h, 8 h, 10 h, 12 h, 24 h) via orbital blood sampling. After the final blood collection, all animals were euthanized by cervical dislocation. The collected blood samples were centrifuged at 4 °C and 3000 rpm for 10 min to obtain approximately 150 μL of plasma. A volume of 100 μL of plasma was precisely transferred, and 30 μL of puerarin (internal standard, 1 μg/mL) and 750 μL of methanol were added to precipitate proteins. After vortex mixing for 1 min, the mixture was centrifuged at 13,000 rpm for 10 min. The supernatant was collected, dried under a gentle stream of nitrogen at 37 °C, and the residue was reconstituted in 150 μL of methanol. The solution was vortexed for 1 min and centrifuged again at 13,000 rpm for 10 min. Finally, the supernatant was transferred into an insert vial for subsequent analysis.

### 2.19. Sample Testing Conditions

The samples were analyzed using a Shimadzu LCMS-8045 triple quadrupole liquid chromatography-mass spectrometry (LC-MS/MS) system (Shimadzu Corporation, Kyoto, Japan). The in vivo pharmacokinetic methodology study of BA-CGA@ nanoparticles can be found in Section S3 of the Supplementary Materials.

Liquid chromatography conditions: A Waters Acquity UPLC HSS T3 column (100 mm × 2.1 mm, 1.7 μm, Waters Corporation, Milford, MA, USA) was used with the column temperature maintained at 35 °C. The mobile phase consisted of 0.1% formic acid in water (A) and acetonitrile (B) under gradient elution at a flow rate of 0.28 mL/min. The injection volume was 4 μL. The gradient program was as follows: 0–0.5 min, 95% A → 85% A; 0.5–1 min, 85% A → 81% A; 1–3 min, 81% A → 78% A; 3–4.5 min, 78% A → 65% A; 4.5–6 min, 65% A → 52% A; 6–7 min, 52% A → 48% A; 7–9 min, 48% A → 5% A; 9–12 min, 5% A → 95% A; 12–13 min, 95% A.

Mass spectrometry conditions: The ion source was an electrospray ionization (ESI) source with a capillary voltage of 3 kV and an ion source temperature of 150 °C. Nitrogen was used as the spray and desolvation gas with a desolvation gas flow of 1000 L·h^−1^ and a desolvation temperature of 600 °C. Detection was performed in multiple reaction monitoring (MRM) mode. For BA, the monitored ion transition was *m*/*z* 445 → 269, with a first quadrupole voltage (Q1) of 21 V, a collision energy (CE) of 20 eV, and a second quadrupole voltage (Q3) of 20 V.

### 2.20. Pharmaceutical Efficacy Verification

The dose of BA (31.13 mg/kg) was derived from the clinically recommended dosage of Pudilan Oral Liquid [33], based on the BA content quantified in our preliminary analysis and converted to a rat equivalent dose. As this study was inspired by the BA-CGA compatibility in PDL, the chosen dose was intended to reflect a clinically relevant exposure level. To allow a direct comparison of pharmacokinetic behavior and anti-inflammatory efficacy attributable to the nano-formulation rather than to differential dosing, the CGA group and the BA-CGA@NPs group were administered at the same dose level (31.13 mg/kg). Aspirin (20 mg/kg) served as a positive control, as this dose is widely established in rodent anti-inflammatory studies [34].

The rats were randomly divided into six groups (*n* = 6 per group): control group, model group, aspirin positive-control group (20.00 mg/kg), CGA group (31.13 mg/kg), BA group (31.13 mg/kg), and BA-CGA@NPs group (31.13 mg/kg). Based on previous studies [14], the AP model was established by topical spraying of 15% ammonia solution (90 μL per spray) into the pharyngeal region twice daily for three consecutive days (days 0–2). On day 4, treatment groups received the corresponding formulations via oral gavage once daily for five days. The positive-control group was administered aspirin by gavage. The other groups received an equal volume of normal saline via intragastric administration. All rats were housed in a controlled environment (25 ± 1 °C; 12 h/12 h light/dark cycle) with free access to food and water.

Following the treatment period, animals were anesthetized with 2% sodium pentobarbital (40 mg/kg, intraperitoneal injection). Blood samples were then collected from the abdominal aorta, after which the animals were euthanized by cervical dislocation, and pharyngeal mucosal tissue samples were harvested. The samples were divided into two portions. One portion was subjected to hematoxylin–eosin (H&E) staining to evaluate the protective effect of BA-CGA@NPs against ammonia-induced AP in rats. Freshly isolated pharyngeal tissues were fixed in 10% paraformaldehyde solution, followed by dehydration through a graded ethanol series and clearing with xylene. After dehydration, tissues were embedded in paraffin and sectioned at a thickness of 4 μm. The sections were deparaffinized, rinsed with ethanol and distilled water, and subjected to conventional H&E staining. Finally, the stained sections were further evaluated for histopathological changes.

The remaining pharyngeal tissues were homogenized in normal saline at a 1:9 (*w*/*v*) ratio. After homogenization, the supernatant was carefully collected and centrifuged at 3000 rpm for 10 min, and the resulting supernatant was retained. The concentrations of IL-1*β*, TNF-*α*, IL-6, and IL-10 were quantified using ELISA kits. The OD at 450 nm was measured with a microplate reader. The concentrations of IL-1*β*, TNF-*α*, IL-6, and IL-10 in the samples were calculated based on their respective standard curves.

### 2.21. Western Blot (WB)

RAW264.7 cells were seeded into 6-well plates at a density of 6 × 10^5^ cells per well, with 2 mL of culture medium per well, and cultured in an incubator for 24 h. The medium was then discarded. Except for the control group, which received serum-free medium, all other groups were treated with 0.5 μg/mL LPS solution prepared in serum-free medium. After 24 h of LPS induction, the medium in the treatment groups (BA, CGA, and BA-CGA@NPs) was replaced with the corresponding drug solutions at a concentration of 50 μg/mL, while the control and model groups received fresh serum-free medium. Following an additional 24 h of incubation, cell lysates were prepared by treating the samples with RIPA buffer (Beijing Solarbio Science & Technology Co., Ltd., Beijing, China, Lot No.: 2500070007) supplemented with a protease inhibitor (PMSF, Solarbio, Lot No.: 20240814). After adjusting the protein content of the samples to a standardized concentration, they were mixed with loading buffer. Due to the specific characteristics of the Toll-like receptor 4 (TLR4) protein, a portion of the samples was heat-denatured at 60 °C for 10 min, while the remainder was heat-denatured at 100 °C for 10 min. The denatured proteins were then separated by sodium dodecyl sulfate-polyacrylamide gel electrophoresis (SDS-PAGE) using a commercial kit (One-Step PAGE Gel Fast Preparation Kit (10%), Cat. E303-01, Vazyme Biotech Co., Ltd., Nanjing, Jiangsu, China). The separated protein bands were electrotransferred onto polyvinylidene fluoride (PVDF) membranes. Target proteins were probed by incubation with specific antibodies, including TLR4 (Zenbio, Chengdu, Sichuan, China, rabbit anti, dilution 1:1000), MyD88 (Zenbio, rabbit anti, dilution 1:1000), NF-*κ*B (Zenbio, rabbit anti, dilution 1:2000), p-NF-*κ*B (Zenbio, rabbit anti, dilution 1:1000), I*κ*B*α* (Zenbio, rabbit anti, dilution 1:1000), p-I*κ*B*α* (Zenbio, rabbit anti, dilution 1:1000), and *β*-actin (Wuhan Servicebio Technology Co., Ltd., Wuhan, Hubei, China, rabbit anti, dilution 1:5000). After washing, the membrane was incubated with HRP-conjugated goat anti-rabbit secondary antibody (Servicebio, dilution 1:10,000). Immunoreactive bands were visualized using a chemiluminescence detection system (Tanon 5200, Tanon Science & Technology Co., Ltd., Shanghai, China).

### 2.22. Statistical Analysis

The data were organized using Microsoft Excel 2019, analyzed and plotted using Origin 2025, and statistically analyzed using GraphPad Prism 8.0. All in vitro experiments were independently performed three times, and the mean values of technical replicates from each independent experiment were used for statistical analysis (*n* = 3). Due to the limited number of independent biological replicates (*n* = 3), formal normality testing was not performed because normality tests have limited statistical power under small sample conditions. Instead, the applicability of parametric statistical analyses was evaluated based on the experimental design, data characteristics, and results of variance homogeneity assessment. Variance homogeneity was evaluated using Bartlett’s test and the Brown–Forsythe test, and the results supported the application of parametric statistical methods. For single-factor experiments, one-way analysis of variance (one-way ANOVA) followed by Dunnett’s multiple comparisons test was performed. For two-factor experiments, two-way analysis of variance (two-way ANOVA) followed by Šídák’s multiple comparisons test was applied. Comparisons between two groups were performed using an independent-samples *t*-test. *p*-value < 0.05 was considered statistically significant.

## 3. Results and Discussion

### 3.1. The Discovery of Self-Assembly Phenomenon and MDS

**Figure 2:**
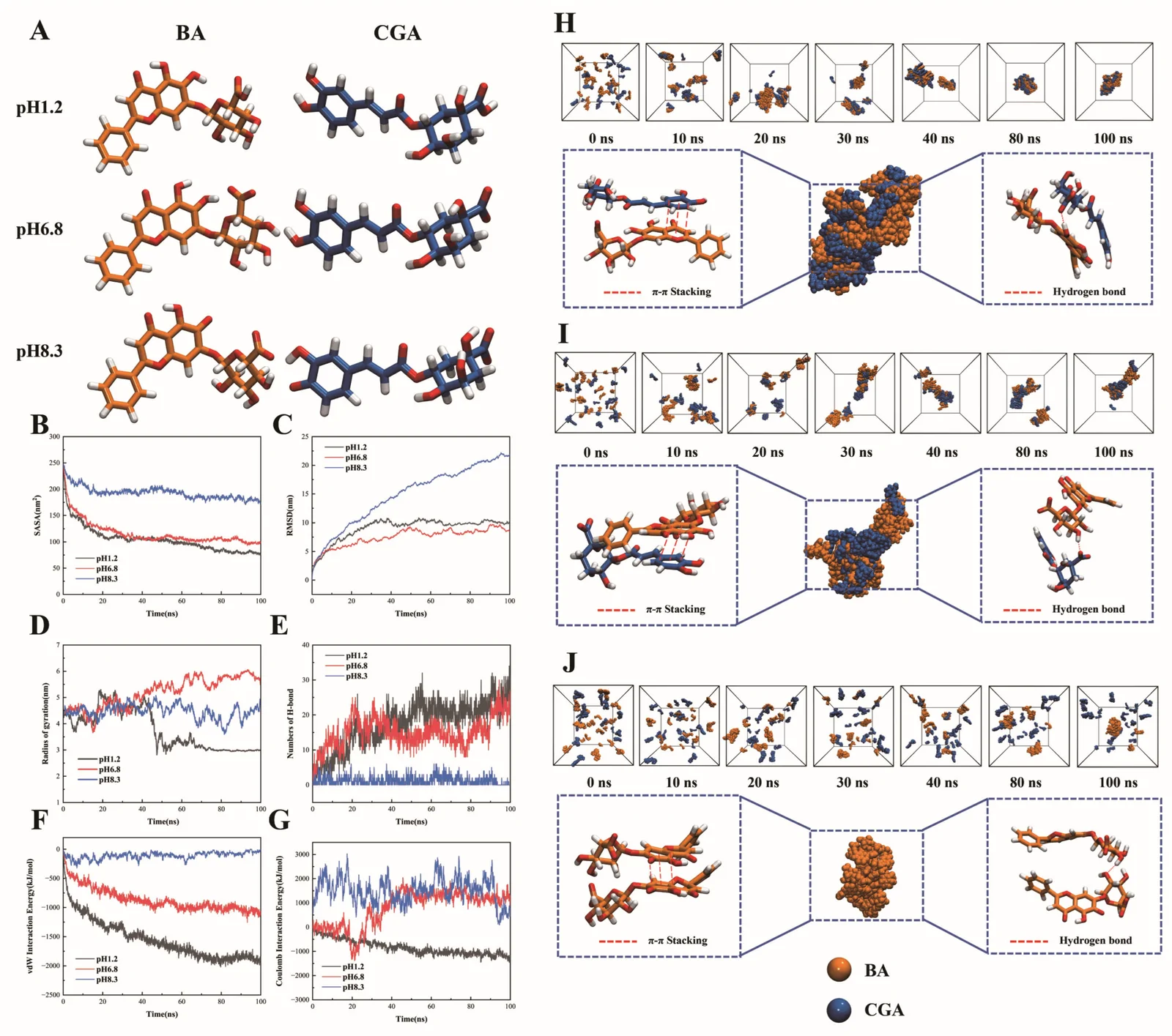
Simulation of BA and CGA assembly characteristics under different pH conditions by molecular dynamics simulation (MDS). (**A**) Molecular structures of BA and CGA and their representative snapshots at pH 1.2, 6.8, and 8.3; (**B**–**G**) time evolution of SASA, RMSD, Rg, number of hydrogen bonds, vdW interaction energy, and Coulombic interaction energy over the course of the simulation; (**H**–**J**) schematic illustrations of the self-assembly MDSs and key interaction sites of the BA-CGA complex at pH 1.2, 6.8, and 8.3.

We first observed that BA and CGA could spontaneously form nanocomplexes in the Pudilan Oral Liquid, as evidenced by the presence of a distinct Tyndall effect (Figure S1A) [35]. Based on this finding, we subsequently prepared the nanocomplexes and preliminarily determined their size distribution (Figure S1B), indicating colloidal aggregation between the two components. This phenomenon motivated us to investigate their intermolecular interactions at the atomic level. To investigate whether BA and CGA could spontaneously self-assemble at the atomic level, MDSs were performed with the two molecules placed at an equimolar ratio in a cubic box. The molecular forms of BA and CGA at different pH values are shown in Figure 2A. During the 100 ns MDSs, the BA-CGA complex system exhibited markedly different aggregation behavior and conformational evolution under different pH conditions (Figure 2C,H–J). At pH 1.2, both BA and CGA existed in their neutral forms. The randomly dispersed molecules gradually formed two clusters within 40 ns, which completely merged into a single, compact, and ordered aggregate before 80 ns. No significant dissociation or free molecules were observed in the subsequent simulation, indicating that acidic conditions effectively promote the rapid self-assembly of BA and CGA. The root-mean-square deviation (RMSD) reached a stable plateau at approximately 45 ns, demonstrating that the system maintained a stable conformation after aggregation.

In contrast, at pH 6.8, the system also formed two independent clusters; however, these clusters remained separated for an extended period and underwent slow fusion only between 80 and 100 ns, indicating that the overall aggregation process was slowed to some extent by electrostatic repulsion. The RMSD stabilized after approximately 50 ns and reached the lowest equilibrium value among the tested conditions, suggesting that the formed aggregates exhibited minimal internal conformational fluctuations and possessed high local structural stability and conformational rigidity. Compared with the rapid and compact aggregation observed at pH 1.2, the pH 6.8 system displayed more moderate aggregation kinetics while still maintaining a stable molecular arrangement.

At pH 8.3, strong electrostatic repulsion significantly inhibited molecular aggregation. Throughout the simulation, only fragmented small clusters were formed; most CGA molecules remained dispersed, while BA underwent limited homomolecular self-aggregation, forming localized enriched regions. The RMSD increased continuously without reaching a plateau, indicating that the system remained in a state of persistent dynamic rearrangement.

The radius of gyration (Rg) results (Figure 2D) showed that the pH 1.2 system formed the most compact aggregate structure. At pH 6.8, the Rg was relatively higher owing to the prolonged coexistence of two clusters; however, it fluctuated only slightly after reaching equilibrium, indicating that the system maintained good local conformational stability. At pH 8.3, the Rg fluctuated continuously around its initial value, reflecting only limited local aggregation. The solvent-accessible surface area (SASA) (Figure 2B) exhibited a trend largely consistent with that of Rg. The pH 1.2 system maintained the lowest SASA after forming a complete aggregate. At pH 6.8, the SASA was slightly higher than that at pH 1.2 due to the presence of two clusters, but it remained stable in the later stage of the simulation. At pH 8.3, most CGA molecules remained exposed to the solvent throughout the simulation, resulting in the highest SASA.

Non-bonded interaction analysis (Figure 2F,G) revealed that the van der Waals (vdW) interaction intensity followed the order pH 1.2 > pH 6.8 > pH 8.3, while the Coulombic interaction shifted from electrostatic attraction at pH 1.2 to electrostatic repulsion at pH 6.8 and pH 8.3, with the strongest repulsion observed at pH 8.3. At pH 1.2, the strong vdW attraction and favorable electrostatic interactions jointly promoted rapid and compact aggregation. At pH 6.8, moderate vdW attraction was counterbalanced by electrostatic repulsion, while hydrogen bonding and π-π stacking maintained a stable molecular arrangement within the aggregates, enabling the system to preserve high local stability without undergoing excessively rapid aggregation. At pH 8.3, strong electrostatic repulsion and competitive solvent interactions rendered stable hydrogen bonds between BA and CGA virtually unattainable; only limited homomolecular self-aggregation of BA occurred via π-π stacking and vdW interactions (Figure 2E). Collectively, these results indicate that an acidic environment is more favorable for driving the rapid self-assembly of BA and CGA, whereas the aggregates formed under near-neutral conditions exhibit higher local conformational stability. As the pH further increases, electrostatic repulsion arising from progressive deprotonation gradually diminishes the self-assembly capacity of the two components.

### 3.2. Morphological Characteristics and Gastrointestinal Stability Study of BA-CGA@NPs

**Figure 3:**
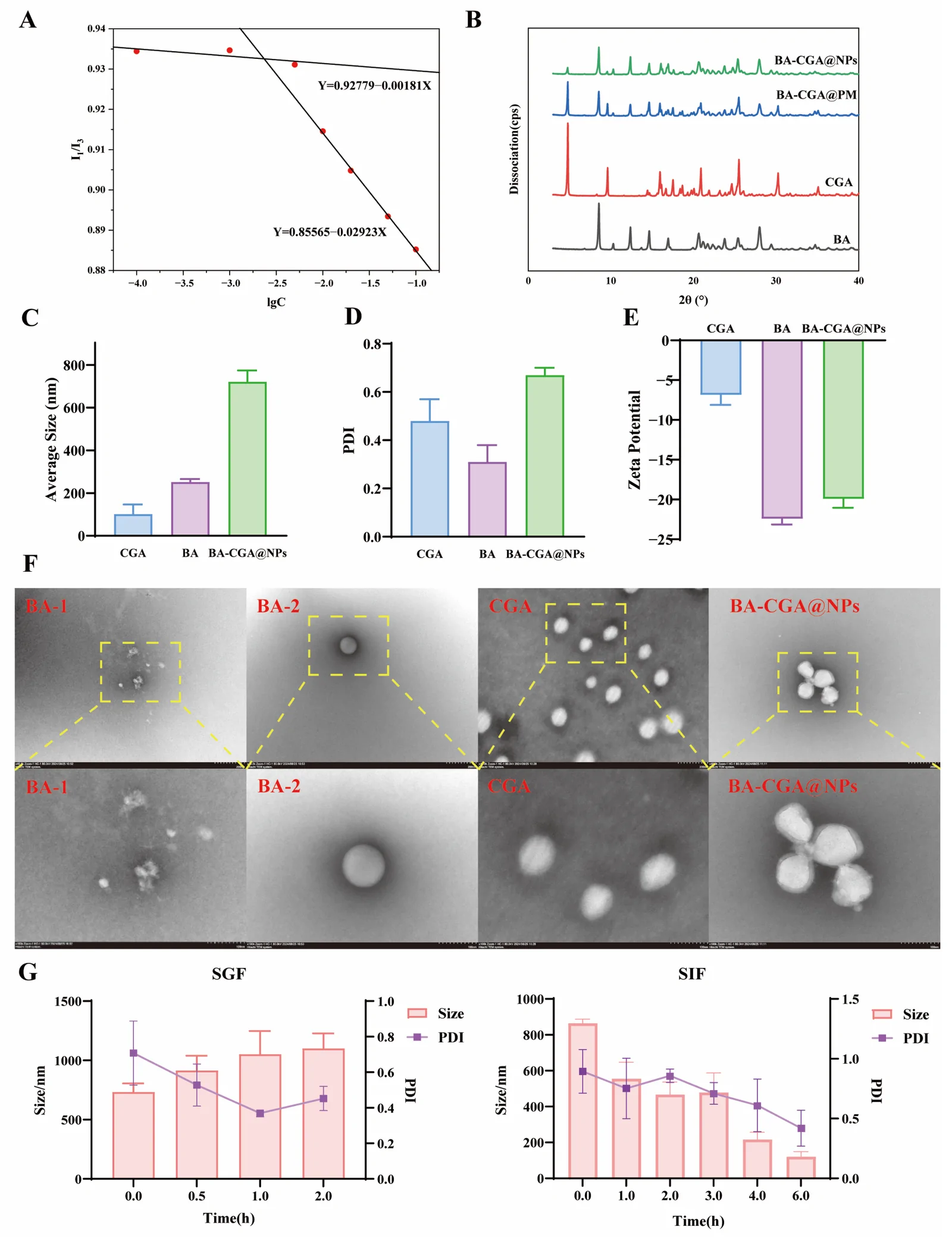
Characterization of BA-CGA@NPs. (**A**) Critical micelle concentration (CMC) of BA-CGA@NPs; (**B**) X-ray diffraction (XRD) patterns of CGA, BA, BA-CGA@NPs and BA-CGA@PM; (**C**–**E**) average size, PDI, and zeta potential of CGA, BA, and BA-CGA@NPs (bar graphs represent mean ± SD, *n* = 3); (**F**) Transmission electron microscopy (TEM) analysis of BA, CGA, and BA-CGA@NPs; (**G**) the particle size and PDI of BA-CGA@NPs in the environment of SGF and SIF.

HPLC quantification revealed that the actual molar ratio of BA to CGA in the lyophilized BA-CGA@NPs was approximately 1.04:1.00, confirming that the two components co-assembled in a near-equimolar stoichiometry consistent with the initial feed ratio. A representative chromatogram is shown in Figure S2. Detailed method validation results, including assessments of precision, repeatability, and stability, are provided in Section S2 of the Supplementary Materials (Tables S1 and S2). The CMC determined using the pyrene fluorescence probe also provided validation. Two linear plots were generated (Y = 0.92779 − 0.00181X and Y = 0.85565 − 0.02923X), which intersected at log C = −2.63, corresponding to a concentration of 2.34 × 10^−3^ mg/mL (Figure 3A). XRD patterns provided information on the crystallinity and structural characteristics of each sample. As shown in Figure 3B, compared with BA, CGA, and BA-CGA@PM, the peak intensities of BA-CGA@NPs decreased significantly, with the first characteristic peak representing CGA notably reduced. This indicates that the formation of BA-CGA@NPs weakened the crystalline order of both components. At the same time, characteristic crystal peaks of BA and CGA were still present in BA-CGA@NPs, suggesting that the formation of BA-CGA@NPs did not alter the crystal forms of BA or CGA, and that the two components interact mainly through non-covalent forces.

DLS was used to investigate the sizes of BA, CGA, and BA-CGA@NPs. The results revealed that the average particle size of BA-CGA@NPs was significantly larger than that of BA and CGA, reaching approximately 720 nm (Figure 3C). Combined with the MDS results, this may be attributed to the extensive molecular aggregation within the system. Meanwhile, the presence of free molecular clusters increased the PDI of the system, indicating a certain variation in the sizes of individual molecular assemblies (Figure 3D). Zeta potential measurements showed that the formation of BA-CGA@NPs significantly enhanced the stability of CGA (Figure 3E). The DLS measurements in aqueous medium revealed a mean hydrodynamic diameter of ~780 nm with a PDI of 0.67 and a zeta potential of approximately −20 mV. While these parameters suggested a relatively broad size distribution and moderate colloidal stability, subsequent experiments in simulated gastrointestinal fluids demonstrated that this initial state reflects pH-dependent aggregation-disassembly rather than inherent instability.

The morphology of BA-CGA@NPs was further examined by TEM. Both CGA and BA exhibited spherical shapes, whereas BA-CGA@NPs displayed interconnected, multi-encapsulated particles with an overall increase in particle size (Figure 3F). The TEM analysis results were generally consistent with the MDS findings.

The results of the simulated gastrointestinal stability experiments (Figure 3G) demonstrated that BA-CGA@NPs exhibited a pH-dependent aggregation-disassembly behavior. In simulated gastric fluid (SGF, pH 1.2), the nanocomplex underwent ordered and self-limited aggregation: the mean hydrodynamic diameter increased from approximately 730 nm to ~1100 nm within 2 h, while the polydispersity index (PDI) decreased from 0.70 to 0.35–0.45, suggesting that the aggregation process was ordered rather than attributable to random flocculation. This observation was consistent with the MDS prediction that at gastric pH, BA and CGA molecules undergo rapid and compact aggregation driven by strong vdW attraction and abundant hydrogen bonding.

By contrast, upon dispersion in simulated intestinal fluid (SIF, pH 6.8), the large aggregates progressively disassembled, with the particle size decreasing from 864 nm to approximately 121 nm and the PDI declining from 0.894 to 0.419. The final particle size was close to that observed by TEM, indicating that the intestinal environment promoted the breakup of secondary aggregates and restored a more homogeneous nanoparticle population. This observation is consistent with the MDS, which demonstrated that deprotonation of the carboxyl groups at pH 6.8 introduces electrostatic repulsion that partially counterbalances intermolecular attractive forces. As a result, further cluster fusion becomes less favorable, preventing excessive aggregation and favoring the maintenance of relatively discrete nanoparticle assemblies.

Although the gastric and intestinal conditions were evaluated in separate incubation experiments, the combined data allow us to propose a mechanistic scenario in which BA-CGA@NPs may undergo protective aggregation in the stomach, followed by dissociation in the intestine that regenerates nanoparticles of a size (~120 nm) favorable for epithelial uptake. It should be noted that this sequential transformation is inferred from independent SGF and SIF experiments and has not been directly demonstrated using a continuous dynamic digestion model; the proposed protective role of gastric aggregation against premature degradation therefore remains to be experimentally verified. The pH-dependent shift in intermolecular forces identified by MDS, from concerted attraction at pH 1.2 to counterbalanced attraction and repulsion at pH 6.8, provides a molecular-level explanation for this dynamic transformation. This proposed mechanism offers a plausible basis for the improved oral absorption observed in the pharmacokinetic study.

### 3.3. Study on the Formation Mechanism of BA-CGA@NPs

**Figure 4:**
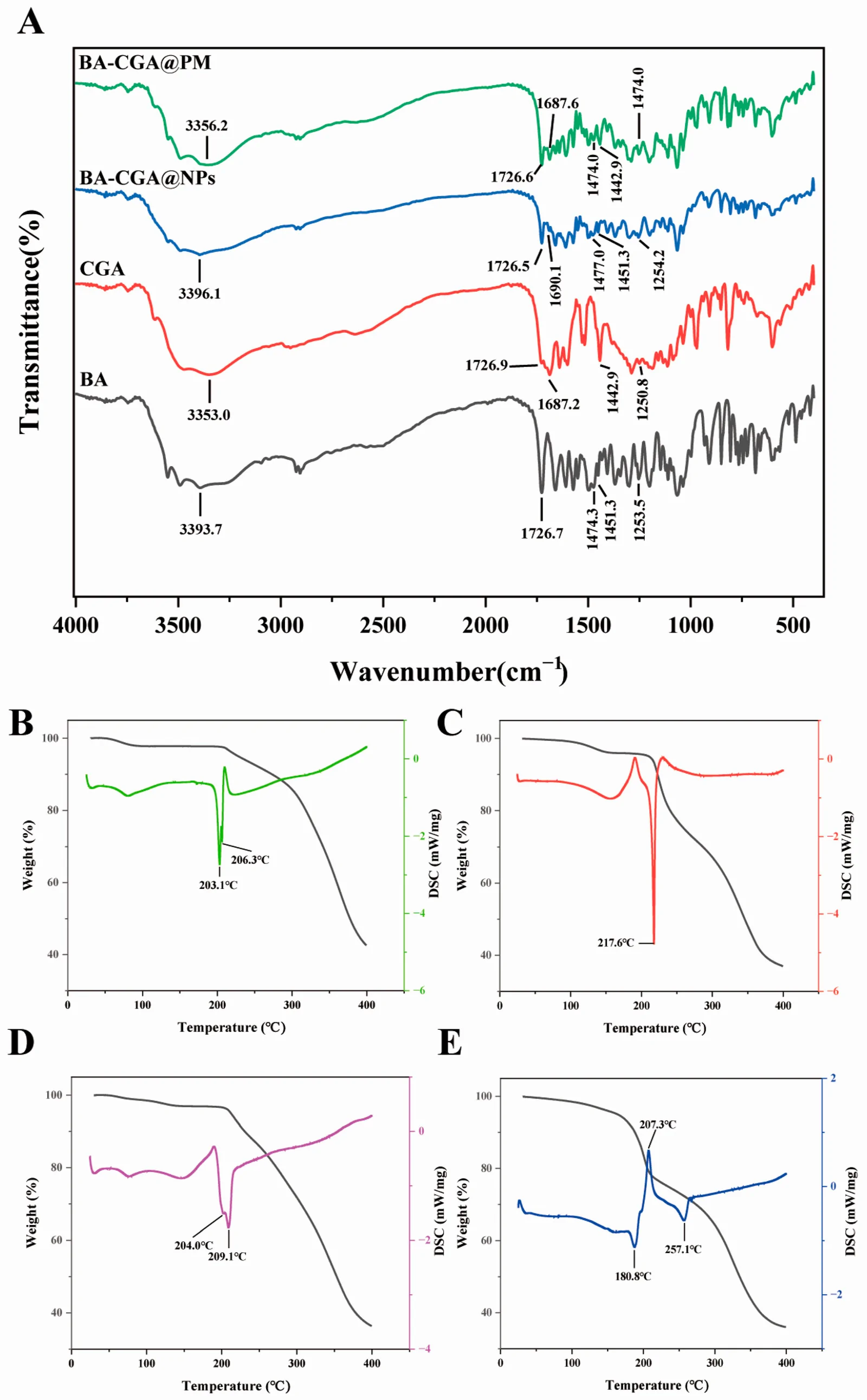
Fourier-transform infrared (FT-IR), Differential scanning calorimetry (DSC), and Thermogravimetric analysis (TGA) characterization. (**A**) FT-IR spectra; (**B**–**E**) DSC and TGA profiles of BA, CGA, BA-CGA@PM, and BA-CGA@NPs.

FT-IR spectroscopy provides information about the functional groups present in the substances (Figure 4A). The spectrum of BA displays various characteristic absorption peaks, including the O-H stretching vibration at 3393 cm^−1^, the C=O stretching vibration at 1726.7 cm^−1^, the aromatic C-H vibration at 1474.3 cm^−1^, the aromatic skeleton vibration at 1451.3 cm^−1^, and the C-O-C stretching vibration at 1253.5 cm^−1^. The spectrum of CGA shows the O-H stretching vibration at 3353 cm^−1^, C=O stretching vibrations at 1726.9 and 1687.2 cm^−1^, the aromatic skeleton vibration at 1442.9 cm^−1^, and the C-O-C stretching vibration at 1250.8 cm^−1^. After the formation of BA-CGA@NPs, the O-H peak in the spectrum shifts to 3396.1 cm^−1^ (blue shift). This change indicates that there is an energy transfer between BA and CGA through hydrogen bonds or π-π stacking interactions, and it can be verified in tandem with the MDS results. Compared to the physical mixture and the individual components, the characteristic peaks for C-O-C stretching, C=O stretching, and aromatic C-H vibrations all exhibit blue shifts in BA-CGA@NPs. These changes suggest the presence of weak intermolecular interactions between BA and CGA, which likely contribute to the stability of BA-CGA@NPs.

TGA and DSC curves provide critical information on the thermal stability and thermal absorption behavior of BA, CGA, and BA-CGA@NPs (Figure 4B–E). The TGA results indicate that, compared to BA and CGA, BA-CGA@PM exhibited mass loss at a lower temperature, suggesting that in the physically mixed state, the overall thermal stability decreases, with reduced resistance to thermal degradation and lower structural integrity. The DSC results show that after the formation of BA-CGA@NPs, the endothermic peak around 180 °C was markedly reduced, and a new exothermic peak emerged around 207 °C, followed by another endothermic event. Combined with the above findings, it is inferred that a portion of BA and CGA did not interact with each other in the system, which is consistent with the unbound molecules observed in the MDS results. This fraction of non-interacting material likely contributed to the endothermic peak at 180 °C. In contrast, the portion that underwent interaction underwent structural changes, leading to alterations in thermal properties and transition temperatures, thereby improving thermal stability, ultimately resulting in an endothermic event at a higher temperature around 257 °C. Understanding the thermal stability and structural changes of such composites is of significant importance for optimizing their performance and stability under various conditions.

### 3.4. The In Vitro Biocompatibility of BA-CGA@NPs

**Figure 5:**
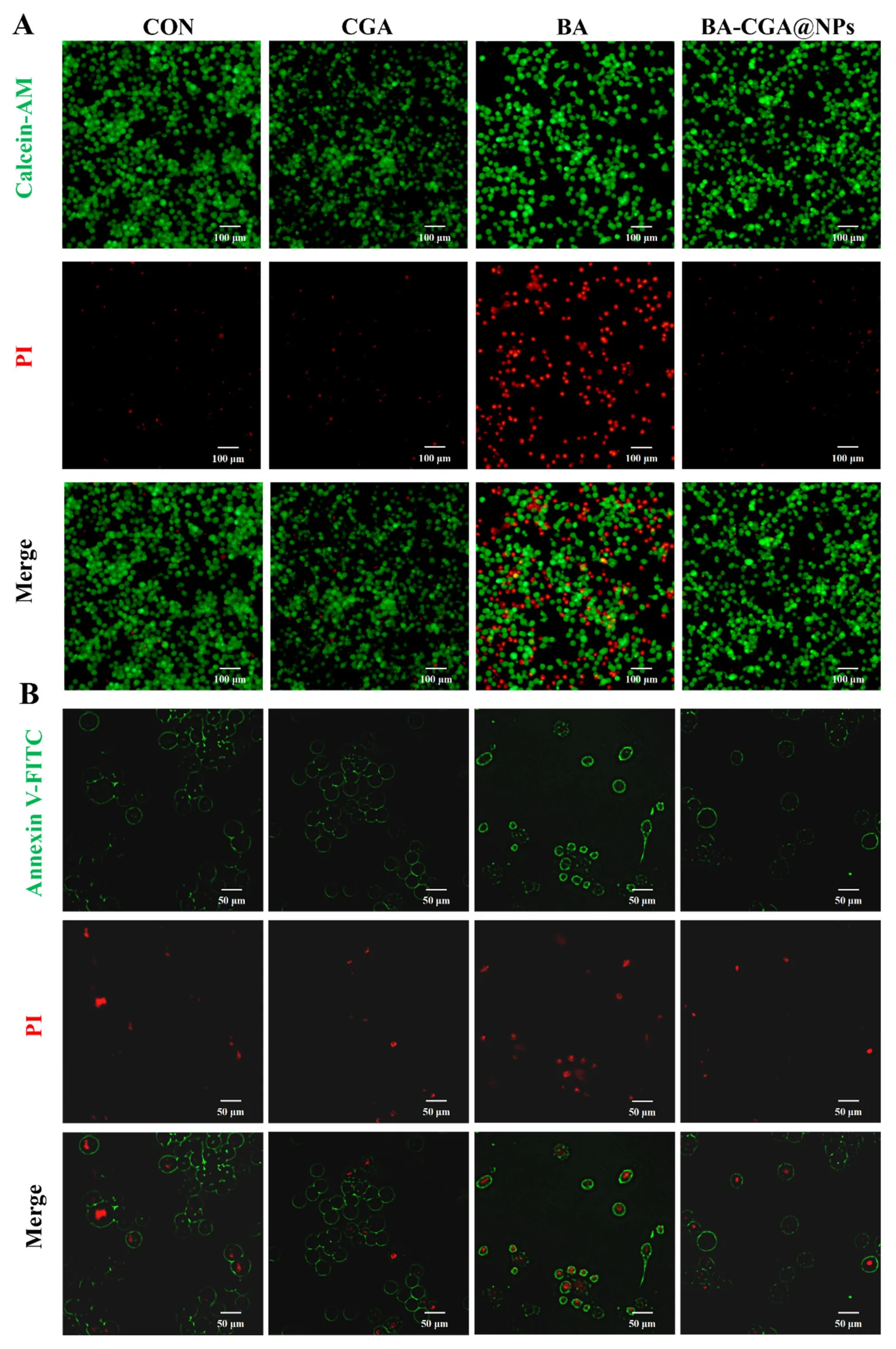
Live/dead cell staining and apoptosis staining of RAW264.7 cells treated with BA, CGA, and BA-CGA@NPs. (**A**) Live/dead cell staining; (**B**) apoptosis staining.

The results from live/dead staining and apoptosis staining together reveal differences in cytocompatibility among BA, CGA, and their self-assembled complex. The live/dead staining results show that BA exhibits relatively high cytotoxicity toward RAW264.7 cells, causing substantial cell death, whereas its toxicity is reduced after self-assembly. This indicates that self-assembly decreases the drug toxicity and leads to better biocompatibility (Figure 5A). The apoptosis staining results further support this conclusion. After Annexin V/PI staining, normal cells show no fluorescence (Annexin V^−^/PI^−^), early-apoptotic cells display green fluorescence (Annexin V^+^/PI^−^), and late-apoptotic or necrotic cells exhibit both green and red fluorescence (Annexin V^+^/PI^+^) [36]. Analysis by Annexin V/PI double staining demonstrated that the apoptosis profile of the CGA group was similar to that of the blank control group, dominated by early apoptosis. In contrast, the BA group showed a higher proportion of late apoptosis and necrosis. The self-assembled complex group presented relatively lower proportions of both apoptotic and necrotic cells, indicating a better cytoprotective effect (Figure 5B). In summary, the complex formed by self-assembly of BA and CGA can significantly reduce the cytotoxicity observed with BA alone, decrease apoptosis and necrosis, and thereby exhibit superior biocompatibility.

### 3.5. Cell Migration

**Figure 6:**
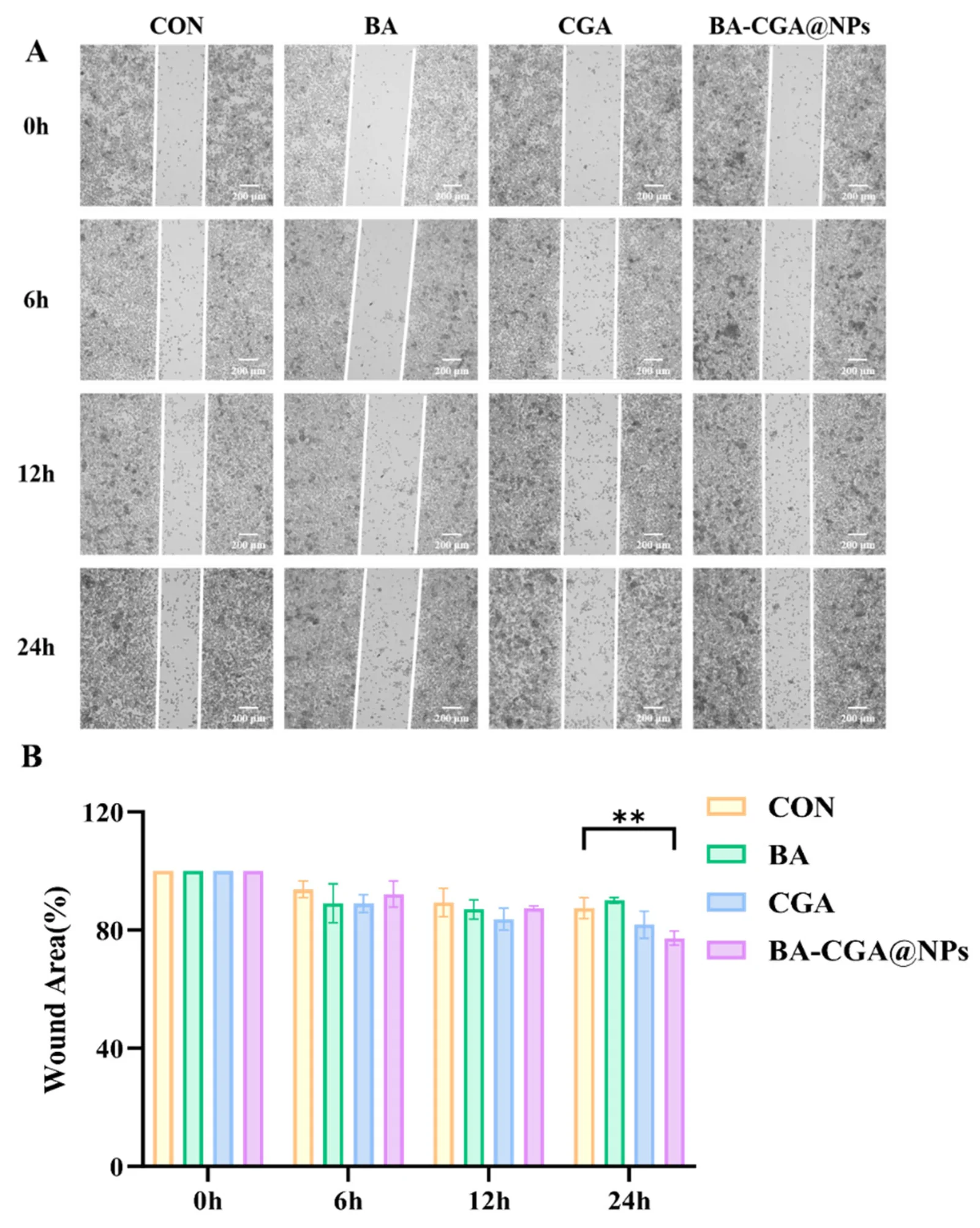
Effects of BA, CGA, and BA-CGA@NPs on the migration ability of RAW264.7 cells. (**A**) Representative images of the scratch wound in RAW264.7 cells at 0 h, 6 h, 12 h, and 24 h after treatment with different formulations. (**B**) Quantified wound area of RAW264.7 cells at 0 h, 6 h, 12 h, and 24 h after treatment (bar graphs represent mean ± SD, *n* = 3). ** *p* < 0.01 vs. control group.

The scratch assay results showed that compared with the blank control group, treatment with BA-CGA@NPs for 24 h significantly promoted wound healing (*p* < 0.01), indicating a strong cell-migration-promoting effect (Figure 6). The CGA treatment group also showed a certain tendency toward wound closure, but the difference was not statistically significant compared with the control group. Notably, the relative wound area in the BA treatment group after 24 h was larger than that in the blank control group. Further analysis suggests that this phenomenon may be related to a certain cyto-inhibitory effect of BA at the tested concentration; although cell viability did not decrease significantly at this concentration, some cells may have altered states or detached, leading to delayed closure of the scratched area.

### 3.6. Effects of BA-CGA@NPs on the Levels of NO, IL-6, TNF-α, and IL-1β in Inflammatory Models

**Figure 7:**
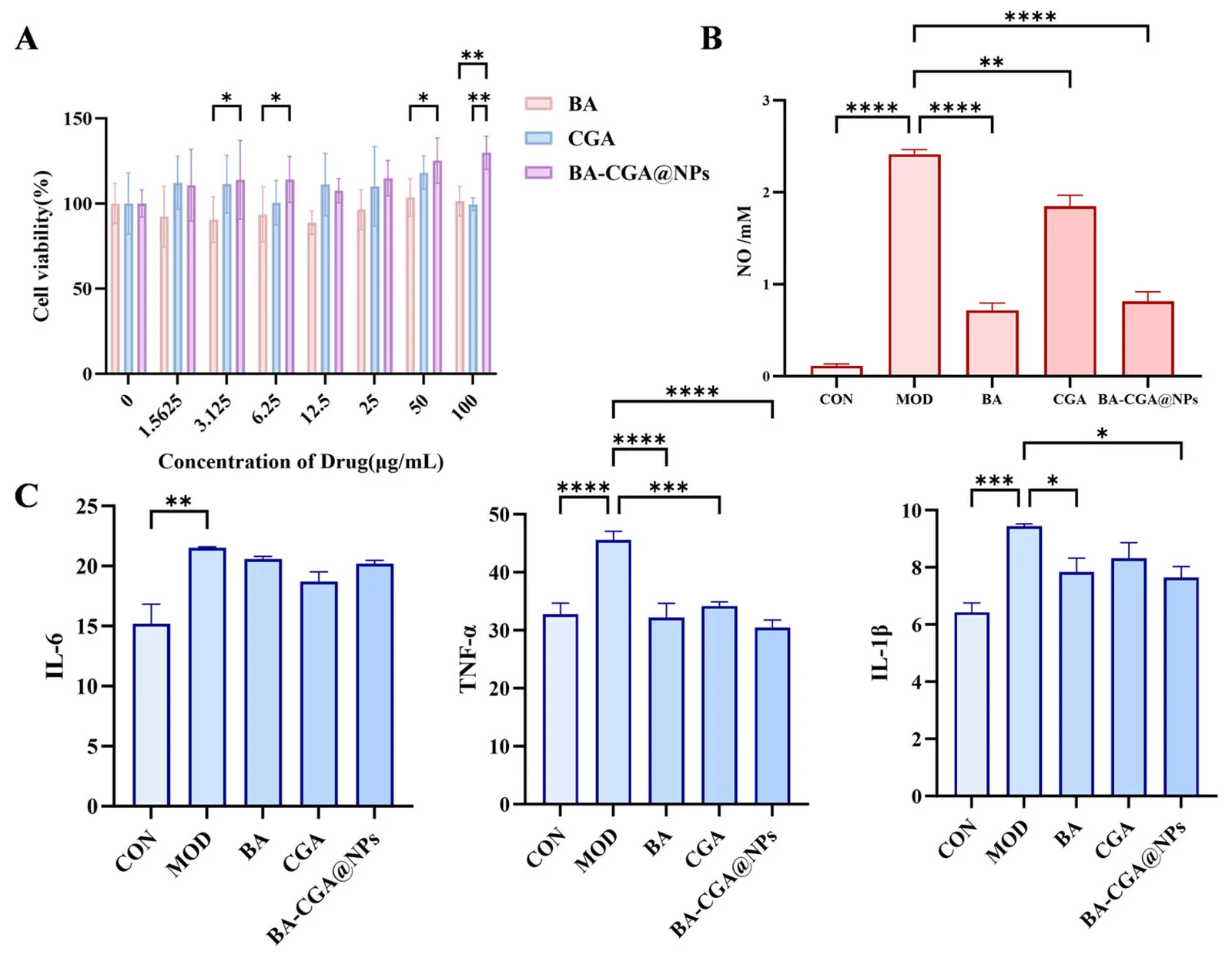
Effects of BA, CGA, and BA-CGA@NPs on cell viability, Nitric oxide (NO) production, and IL-6, TNF-*α*, and IL-1*β* levels in Lipopolysaccharide (LPS)-stimulated RAW264.7 cells. (**A**) Effects of BA, CGA, and BA-CGA@NPs on RAW264.7 cell viability. Bar graphs represent mean ± SD, *n* = 6 (repeat 3 times, with 6 holes each time); * *p* < 0.05, ** *p* < 0.01 vs. the BA-CGA@NPs group. (**B**) Effects of BA, CGA, and BA-CGA@NPs on NO levels in LPS-induced RAW264.7 cells. Bar graphs represent mean ± SD, *n* = 6 (repeat 3 times, with 6 holes each time). (**C**) Effects of BA, CGA, and BA-CGA@NPs on IL-6, TNF-*α*, and IL-1*β* levels in LPS-induced RAW264.7 cells. Bar graphs represent mean ± SD, *n* = 6 (repeat 3 times, with 6 holes each time); * *p* < 0.05, ** *p* < 0.01, *** *p* < 0.001, **** *p* < 0.0001 vs. the model group (applicable to B and C).

The CCK-8 assay was used to evaluate the effects of different concentrations of BA, CGA, and BA-CGA@NPs on the viability of RAW264.7 cells (Figure 7A). A concentration of 50 μg/mL was selected for BA, CGA, and BA-CGA@NPs in subsequent experiments, and this result was consistent with the staining findings in Section 3.4, validating its appropriateness. Measurement of NO levels showed that LPS induction led to a highly significant increase in NO (*p* < 0.0001), while treatment with BA, CGA, and BA-CGA@NPs significantly reduced NO levels (*p* < 0.01) (Figure 7B). Notably, the BA and BA-CGA@NPs groups exhibited extremely significant reductions (*p* < 0.0001), suggesting that both agents prominently suppress excessive NO production during inflammation, likely through the modulation of relevant inflammatory signaling pathways, thereby exerting potential anti-inflammatory effects.

ELISA results for IL-6, TNF-*α*, and IL-1*β* revealed that after LPS stimulation, IL-6, TNF-*α*, and IL-1*β* were elevated to different extents (*p* < 0.01). Following drug treatment, IL-6 showed a decreasing trend; TNF-*α* was significantly reduced in all treated groups (*p* < 0.001), with the BA and BA-CGA@NPs groups showing extremely significant decreases (*p* < 0.0001); IL-1*β* levels also decreased markedly, with the reductions in the BA and BA-CGA@NPs groups reaching statistical significance (*p* < 0.05) (Figure 7C).

In summary, BA, CGA, and their self-assembled complex BA-CGA@NPs demonstrated clear anti-inflammatory activity in the LPS-induced inflammation model. They markedly inhibited excessive NO production and selectively down-regulated key cytokines such as TNF-*α* and IL-1*β*. Overall, BA alone and BA-CGA@NPs exhibited superior effects compared with CGA alone, suggesting that BA may play a leading role in the anti-inflammatory action and that its co-assembly with CGA into nanocomplexes contributes to the improved anti-inflammatory efficacy.

### 3.7. The Influence of BA-CGA@NPs on the Level of ROS

**Figure 8:**
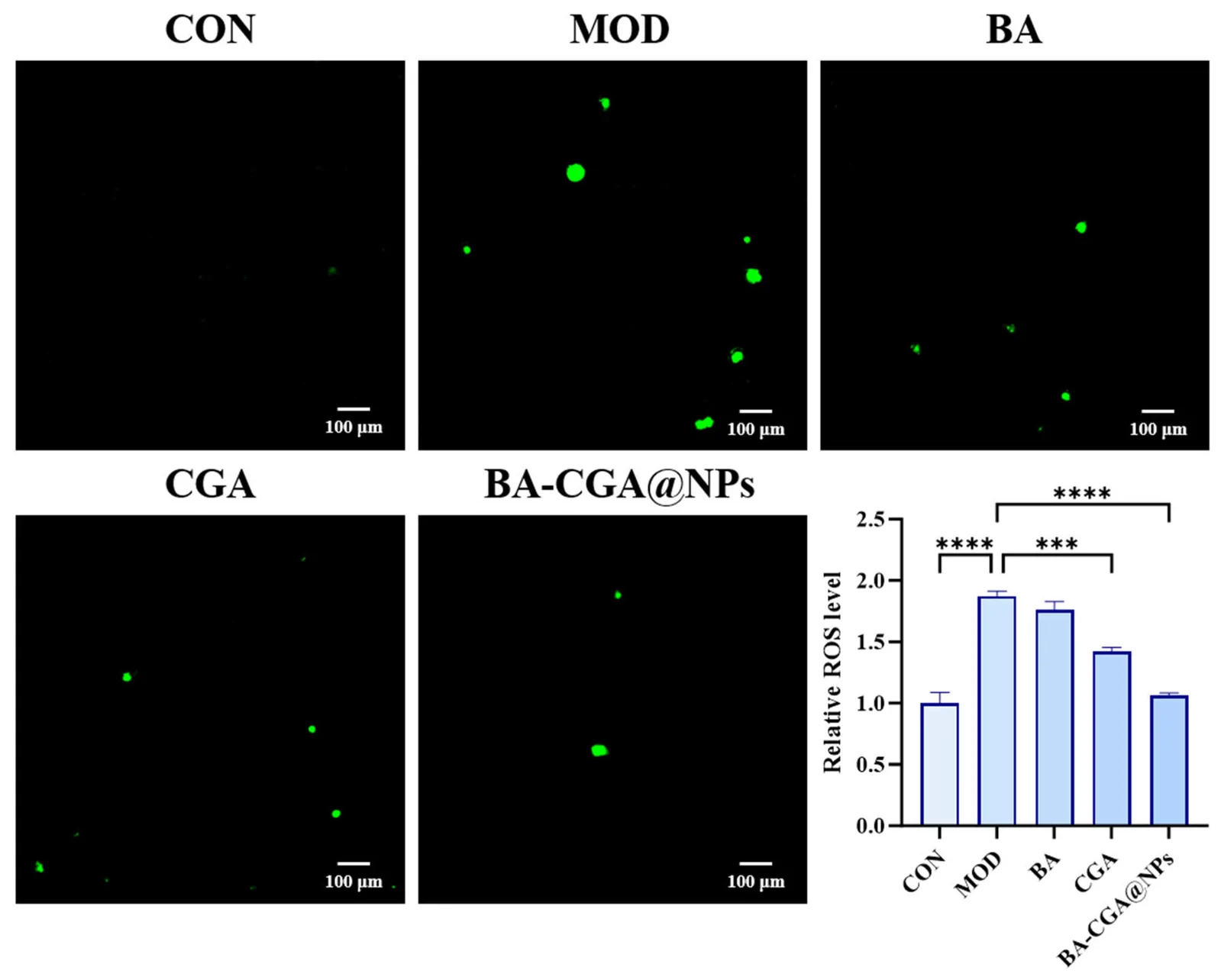
Effects of BA, CGA, and BA-CGA@NPs on Reactive oxygen species (ROS) levels in LPS-induced RAW264.7 cells. Bar graphs represent mean ± SD, *n* = 4; *** *p* < 0.001, **** *p* < 0.0001 vs. the model group.

The results of ROS staining showed that compared with the blank control group, intracellular ROS levels were significantly elevated after LPS stimulation (*p* < 0.0001). All treatment groups reduced the LPS-induced increase in ROS to varying degrees. The CGA group exhibited a highly significant decrease (*p* < 0.001), while the BA-CGA@NP group showed a particularly pronounced reduction (*p* < 0.0001), indicating that BA-CGA@NPs plays a prominent role in scavenging ROS and alleviating oxidative stress (Figure 8).

### 3.8. In Vivo Pharmacokinetic Study and Pharmacodynamic Validation of BA-CGA@NPs

After oral administration to each group of rats, the pharmacokinetics of BA-CGA@NPs and BA were evaluated. Key pharmacokinetic parameters, including the area under the concentration–time curve (*AUC*), mean residence time (MRT), peak plasma concentration (*C*_max_), half-life (t_1/2_), and time to reach *C*_max_ (*T*_max_), are presented in Table 1. The extrapolated percentages of *AUC*_(0–∞)_ were 8.2% and 1.1% for BA and BA-CGA@NPs, respectively, both well below the 20% threshold, confirming the reliability of the terminal phase fitting and supporting the use of observed *AUC*_(0–t)_ as the primary exposure metric. The pharmacokinetic results showed that, compared with the BA-alone group, BA in the form of BA-CGA@NPs exhibited higher systemic exposure (*AUC*_(0–t)_ 91.25 ± 4.20 vs. 74.37 ± 1.11 mg·L^−1^·h, *p* < 0.01), higher *C*_max_ (12.45 ± 0.96 vs. 8.16 ± 0.36 mg/L, *p* < 0.01), shorter MRT, and shorter *t*_1/2_, while the *T*_max_ of the major (secondary) peak was comparable between the two groups. These results indicate that BA-CGA@NPs enhanced the extent of BA oral absorption, as reflected by the increased *C*_max_ and *AUC*_(0–t)_, rather than the absorption rate. Based on *AUC*_(0–t)_, the relative oral bioavailability of BA from the nanocomplex was approximately 1.23-fold that of free BA.

**Figure 9:**
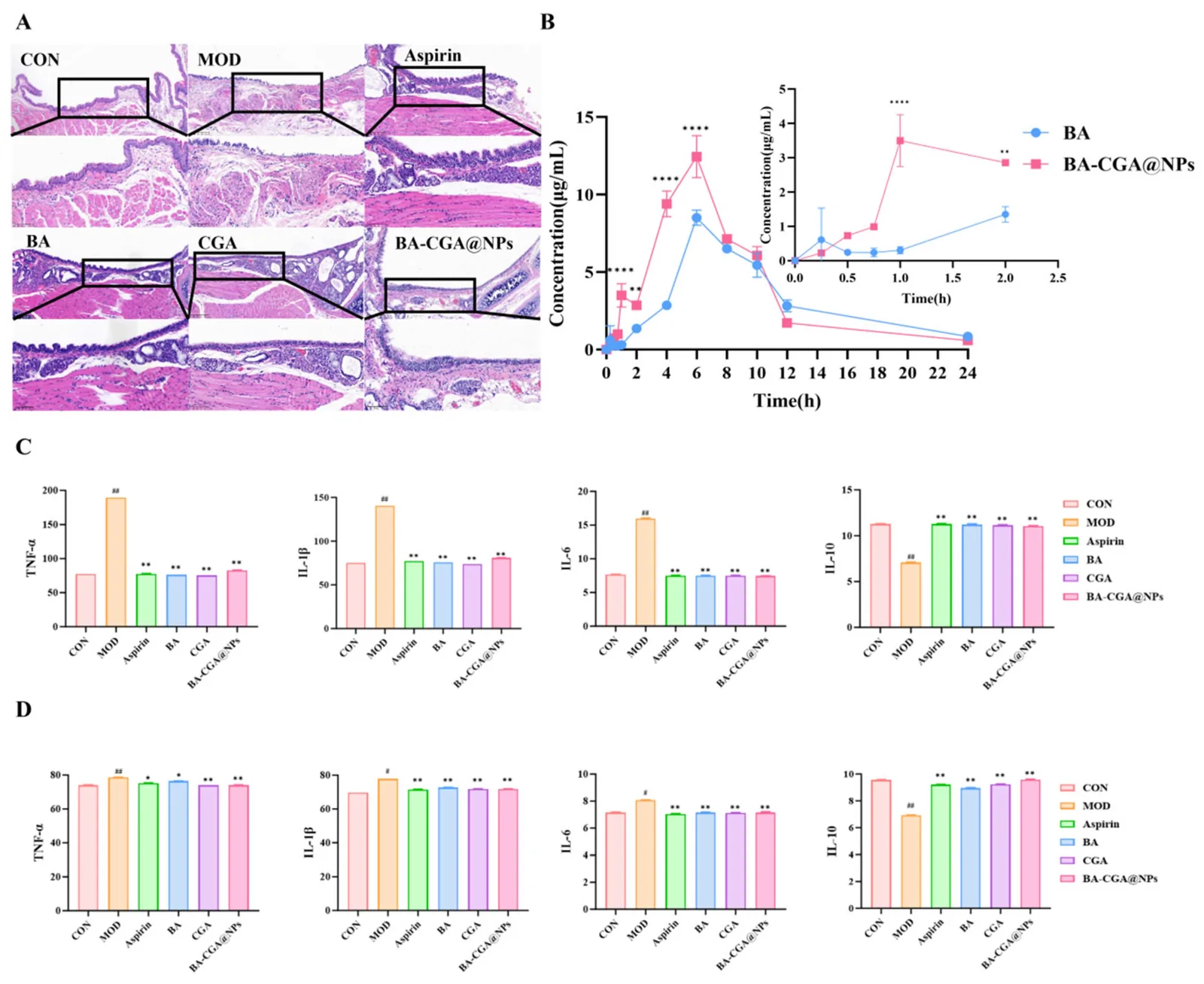
Pharmacokinetics and pharmacodynamics of BA-CGA@NPs. (**A**) H&E staining. (**B**) Plasma concentration–time profile; bar graphs represent mean ± SD, *n* = 6; ** *p* < 0.01, **** *p* < 0.0001 vs. the BA group. (**C**) Inflammatory cytokines in tissue homogenates; (**D**) inflammatory cytokines in serum; bar graphs represent mean ± SD, *n* = 6; ^#^ *p* < 0.05, ^##^ *p* < 0.01 vs. the control group; * *p* < 0.05, ** *p* < 0.01 vs. the model group (applicable to C and D).

The plasma concentration–time profile of orally administered BA frequently displays a double-peak phenomenon (Figure 9B), which is commonly attributed to enterohepatic recirculation or segmental absorption along the gastrointestinal tract. In the BA-CGA@NPs group, the pharmacokinetic profile was altered in a manner consistent with the pH-responsive aggregation-disassembly behavior observed in vitro and predicted by MDS. During the gastric phase, the nanocomplex underwent acid-induced aggregation, which delayed the initial minor absorption peak relative to free BA. Upon entering the intestine, the aggregates dissociated into smaller nanoparticles, thereby facilitating the absorption of intact BA and resulting in a higher second (major) peak concentration (*C*_max_: 12.45 vs. 8.16 mg/L). This *C*_max_ value is also presented in Table 1. The *T*_max_ values of the major peak were comparable between the two groups (7.33 vs. 6.67 h, *p* > 0.05), indicating that the nanocomplex mainly enhanced the extent rather than the rate of BA absorption. The shortened t_1/2_ (2.88 vs. 5.46 h) and reduced MRT suggest accelerated systemic elimination, which may be attributable to altered tissue distribution or enhanced biliary excretion of the nano-formulated BA; the underlying mechanism requires further investigation. Importantly, the substantially elevated *C*_max_ compensated for the faster elimination, yielding a net increase in total BA exposure. All bioavailability conclusions in this study are based on the observed *AUC*_(0–t)_ rather than the extrapolated *AUC*_(0–∞)_. The above analysis methods meet the requirements of relevant bioanalysis method validation guidelines in terms of linearity, precision, accuracy, stability, recovery rate and matrix effect (Tables S3–S6, Section S3 of the Supplementary Materials).

Following the successful establishment of the rat AP model, treatment was administered via oral gavage for 5 days. The morphological changes in pharyngeal tissues across different groups were observed by H&E staining, and the levels of inflammatory factors in serum and tissue homogenates were measured using ELISA kits to evaluate therapeutic efficacy. In the H&E-stained sections, compared with the blank control group, the model group showed almost complete loss of the squamous epithelium (the purple multi-layer cell band at the edge) and severe inflammatory infiltration. In contrast, all treatment groups showed a certain degree of epithelial repair and a reduction in submucosal inflammatory infiltration. Among them, the squamous epithelium repair of BA-CGA@NPs was the most complete, indicating a superior therapeutic effect (Figure 9A).

In the ELISA assays, compared with the blank control group, the model group showed significantly higher levels of IL-1*β*, TNF-*α*, and IL-6 in tissue homogenates (*p* < 0.001), confirming successful modeling and a more severe inflammatory response. In both pharyngeal tissue supernatants and serum, the levels of IL-1*β*, TNF-*α*, and IL-6 showed a decreasing trend in all treatment groups, with statistically significant differences (*p* < 0.05). Compared with the model group, the aspirin group, BA group, CGA group, and BA-CGA@NPs group all exhibited significant reductions in IL-1*β*, TNF-*α*, and IL-6 levels (*p* < 0.001). Conversely, the level of IL-10 was significantly increased compared with the model group (*p* < 0.0001) (Figure 9C,D). Notably, the BA-CGA@NPs group demonstrated superior therapeutic efficacy. These results indicate that the formation of BA-CGA@NPs was associated with improved oral bioavailability of BA, as evidenced by the 1.23-fold increase in *AUC*_(0–t)_ and the 1.53-fold increase in C_max_, and possessed the ability to ameliorate inflammatory cytokine levels in both tissues and serum of rats with AP.

### 3.9. WB Analysis

The TLR4/MyD88/NF-*κ*B signaling pathway is closely associated with the regulation of inflammatory responses [37,38,39]. As a pattern recognition receptor, TLR4 binds to the adaptor protein MyD88 and triggers downstream pro-inflammatory signaling cascades via NF-*κ*B activation [40]. Under normal physiological conditions, the NF-*κ*B complex remains inactive in the cytoplasm by binding to its inhibitor, inhibitor of NF-*κ*B (I*κ*B) [41]. Upon activation, I*κ*B undergoes phosphorylation, leading to the release of NF-*κ*B from I*κ*B. The dissociated NF-*κ*B then translocates into the nucleus and initiates the production of inflammatory mediators [42,43]. Therefore, during AP, activation of NF-*κ*B can drive the expression of pro-inflammatory cytokines and signaling molecules, resulting in sustained inflammation [44,45].

**Figure 10:**
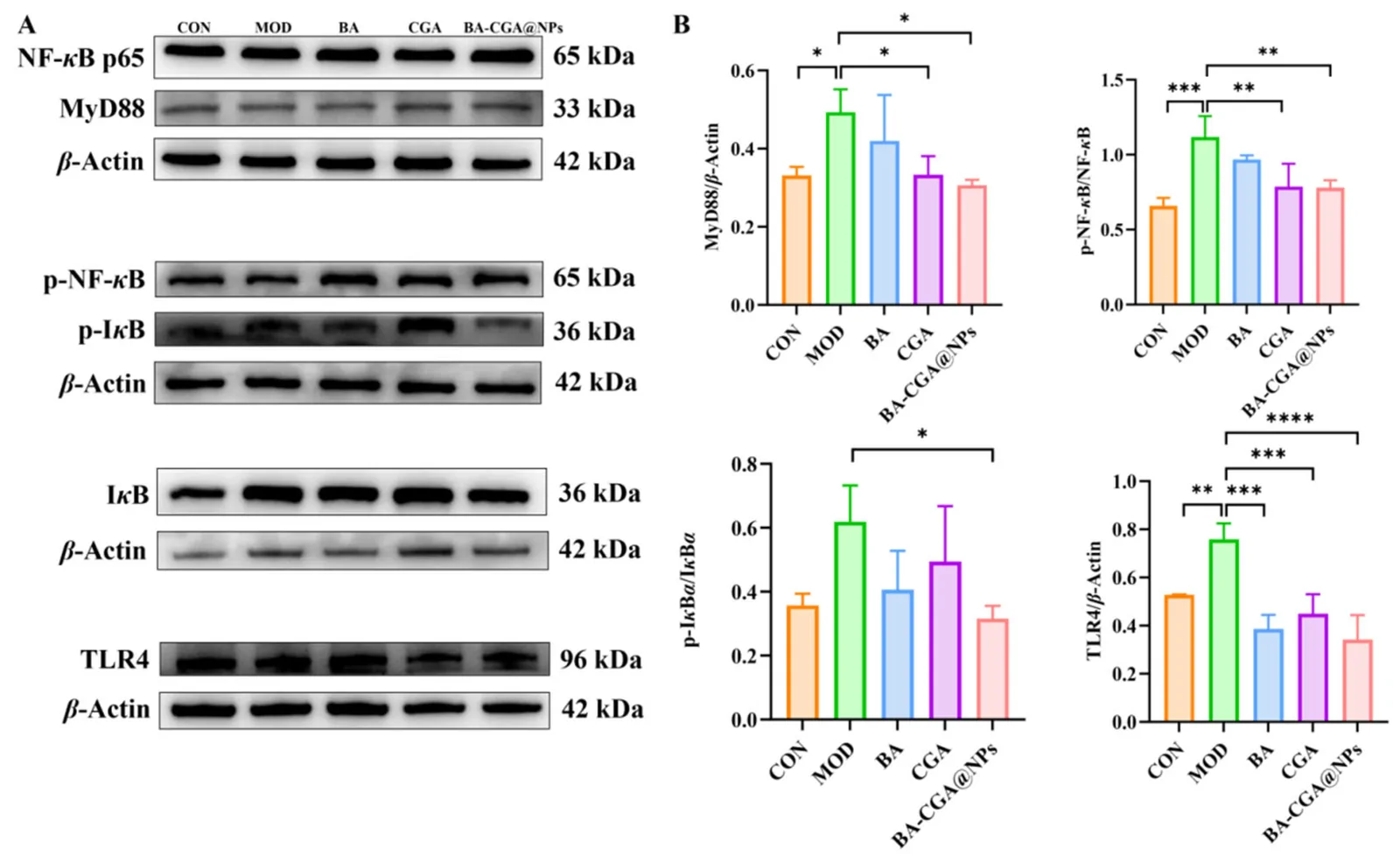
Western blot (WB) analysis. (**A**) WB analysis of the Toll-like receptor 4 (TLR4)/Nuclear factor-κB (NF-*κ*B)/MyD88 pathway; (**B**) quantitative analysis of TLR4/*β*-Actin, MyD88/*β*-Actin, Phosphorylated IκBα (p-I*κ*B*α*)/I*κ*B*α*, and Phosphorylated NF-κB (p-NF-*κ*B)/NF-*κ*B protein expression ratios. Bar graphs represent mean ± SD, *n* = 3; * *p* < 0.05, ** *p* < 0.01, *** *p* < 0.001, **** *p* < 0.0001 vs. the model group.

As shown in Figure 10, the model group displayed significantly elevated protein levels of TLR4 and MyD88 (*p* < 0.05), along with markedly increased expression of phosphorylated NF-*κ*B (p-NF-*κ*B) and phosphorylated I*κ*B*α* (p-I*κ*B*α*) (*p* < 0.05), confirming that LPS stimulation effectively activates this signaling pathway. Treatment with BA, CGA, or BA-CGA@NPs differentially attenuated the aberrant activation of the pathway compared with the model group. Among these interventions, BA-CGA@NPs exerted the most pronounced inhibitory effect, particularly on TLR4 protein expression (*p* < 0.0001), and suppressed downstream effectors including MyD88, p-NF-*κ*B, and p-I*κ*B*α* more effectively than either BA or CGA alone. Two factors may account for this enhanced efficacy. First, the self-assembly of BA and CGA into nanoparticles likely improves their stability and solubility, thereby enhancing their cellular uptake and intracellular availability. Second, the superior inhibition of TLR4 by BA-CGA@NPs suggests that the anti-inflammatory effects of BA-CGA@NPs were associated with the modulation of the TLR4/MyD88/NF-*κ*B signaling pathway.

In summary, the anti-inflammatory effects of BA-CGA@NPs were associated with the modulation of the TLR4/MyD88/NF-*κ*B signaling pathway, as evidenced by reduced protein levels of TLR4 and MyD88 and decreased phosphorylation of NF-*κ*B and I*κ*B*α*, suggesting a possible involvement of this pathway in the transcriptional regulation of downstream inflammatory mediators. These findings are consistent with the anti-inflammatory, antioxidant, and cytoprotective effects observed in cellular models, and provide preliminary evidence, at the molecular level, for a potential mechanism through which BA-CGA@NPs alleviate inflammation. However, the precise molecular mechanism remains to be fully elucidated.

### 3.10. Discussion

Taken together, the pharmacokinetic and pharmacodynamic results indicate that the potent anti-inflammatory efficacy of BA-CGA@NPs in the acute pharyngitis rat model is primarily driven by the nano-formulation-enabled fundamental improvement in the oral absorption of BA. Pharmacokinetic data showed that the systemic exposure of BA following oral administration of BA-CGA@NPs was approximately 1.23-fold higher than that of free BA, providing the determinative material basis for the superior therapeutic outcome. The combined pharmacological action of BA and CGA may contribute to the overall efficacy after the nanocomplex has successfully overcome the absorption barrier and achieved effective delivery to the target site. Therefore, in the efficacy-enhancement mechanism of BA-CGA@NPs, absorption improvement serves as the primary driver, while the co-delivery of both agents to the target site may provide an additional pharmacological benefit that warrants further investigation.

It should be noted that the sample sizes in this study, while consistent with common practice in nanomedicine and compliant with the 3R principle, were relatively modest. Larger sample sizes in future studies would allow more precise estimation of effect sizes and detection of subtle pharmacological interactions.

Under the actual physiological conditions of oral administration, a physical mixture lacking nano-structural stabilization cannot overcome the dissolution and solubility bottlenecks of BA; consequently, its oral absorption behavior is essentially indistinguishable from that of free BA alone. The pharmacokinetic and pharmacodynamic data obtained from the free BA group therefore already indirectly reflect the in vivo performance of the physical mixture. Furthermore, the rapid phase separation and precipitation of the physical mixture in the dosing vehicle would result in a non-uniform gavage suspension, introducing uncontrollable dosing errors and rendering it an unreliable comparator. Nevertheless, we recognize that this design precludes a formal quantitative assessment of the degree of synergy (e.g., combination index) between BA and CGA at the target tissue level. In future work, under conditions where absorption differences are controlled, isobolographic analysis and related methods will be employed to specifically evaluate the synergistic effect of the two agents in pharyngeal tissue, thereby further refining the theoretical basis of this strategy.

Regarding the in vitro study design, a pharmacological positive control (e.g., dexamethasone or a specific NF-*κ*B inhibitor) was not included, as the primary objective was to compare the anti-inflammatory efficacy of BA-CGA@NPs with that of free BA or CGA alone. We acknowledge that the absence of an established anti-inflammatory agent as a positive control limits the independent validation of the assay system, and this will be addressed in future studies. In the in vivo study, aspirin served as the positive control to provide a clinically relevant anti-inflammatory benchmark; the inclusion of a pathway-specific inhibitor may further strengthen the mechanistic conclusion.

It should be noted that the in vivo AP model used in this study was established by topical ammonia irritation, which reproduces chemically induced sterile pharyngeal inflammation rather than streptococcal or viral pharyngitis emphasized in the Introduction. Similarly, the in vitro model employed LPS (a Gram-negative bacterial component) to stimulate RAW264.7 macrophages, which does not fully recapitulate the signaling events triggered by *Streptococcus* or respiratory viruses. These models were selected because the ammonia-induced AP model is a well-established and reproducible system that recapitulates key histopathological features of acute pharyngitis (mucosal congestion, edema, and inflammatory cell infiltration), while the LPS-stimulated macrophage model is a standard platform for evaluating anti-inflammatory mechanisms via the TLR4/NF-*κ*B pathway [14]. Given that the primary objective of this work was to assess the anti-inflammatory efficacy and absorption enhancement of BA-CGA@NPs, these models were considered appropriate for the current proof-of-concept stage. Nevertheless, the efficacy of BA-CGA@NPs in infectious pharyngitis models (e.g., Streptococcus- or virus-induced) warrants dedicated evaluation in future studies to fully establish the translational potential of this nano-formulation under clinically relevant infectious conditions.

Additionally, the dose used in the PK study (200 mg/kg) was higher than that employed in the pharmacodynamic study (31.13 mg/kg). This was necessary because the pharmacodynamic dose resulted in plasma BA concentrations near or below the quantification limit in the free BA group, preventing reliable pharmacokinetic modeling. The improved absorption profile of BA-CGA@NPs observed at the PK dose (1.23-fold higher *AUC*_(0–t)_ and 1.53-fold higher *C*_max_) is consistent with the enhanced therapeutic efficacy at the pharmacodynamic dose, as evidenced by the significantly greater reductions in serum and pharyngeal tissue levels of TNF-*α*, IL-1*β*, and IL-6 in the BA-CGA@NPs group compared with free BA alone, along with marked improvement in pharyngeal histopathology. While the PK and PD studies were conducted at different doses, the superior oral absorption conferred by the nano-formulation provides a plausible pharmacokinetic basis for the improved anti-inflammatory outcomes.

Furthermore, it should be noted that the gastric and intestinal stability assays were performed by directly dispersing BA-CGA@NPs into each medium independently, rather than in a sequential SGF-to-SIF transfer model. While this design effectively isolated the pH-specific effects on nanoparticle behavior, future studies employing a continuous dynamic digestion model would provide additional insights into the sequential transformation of the nanocomplex along the gastrointestinal tract.

## 4. Conclusions

This study successfully constructed and systematically characterized a nanocomplex (BA-CGA@NPs) formed via supramolecular self-assembly of BA and CGA. MDS and multiple spectroscopic characterizations confirmed that the assembly is primarily driven by non-covalent interactions, such as intermolecular hydrogen bonding and electrostatic forces, resulting in a nano-system. This assembly strategy was associated with improved biocompatibility and enhanced oral bioavailability of BA, as evidenced by the 1.23-fold increase in *AUC*_(0–t)_ and the 1.53-fold increase in *C*_max_.

In vitro studies demonstrated that, compared with the individual components, BA-CGA@NPs exhibited superior biocompatibility and more effectively suppressed the LPS-induced inflammatory response in RAW264.7 macrophage models. This included reducing the levels of NO, ROS, and key pro-inflammatory cytokines (TNF-*α*, IL-1*β*), while also promoting cell migration and repair.

Key pharmacokinetic evaluations demonstrated that oral administration of BA-CGA@NPs was associated with increased systemic exposure of BA, as evidenced by a 1.53-fold higher *C*_max_ and a 1.23-fold greater *AUC_(_*_0–t*)*_, which may contribute to the enhanced efficacy. In a rat model of AP, treatment with BA-CGA@NPs effectively ameliorated histopathological damage in pharyngeal tissues and significantly reduced the levels of inflammatory cytokines (IL-1*β*, TNF-*α*, IL-6) in both serum and tissues, with overall therapeutic efficacy superior to that of BA or CGA alone. Mechanistic studies further revealed that its anti-inflammatory effect was closely associated with the modulation of the TLR4/MyD88/NF-*κ*B signaling pathway, as reflected by the reduced protein levels of key pathway components.

In summary, this study presents a simple and green nano-assembly strategy. By constructing a supramolecular nanocomplex from BA and CGA, the oral bioavailability and biocompatibility were effectively improved. The markedly enhanced anti-AP efficacy observed both in vitro and in vivo is primarily driven by this nanocomplex-enabled absorption breakthrough, upon which the superimposed pharmacodynamic contributions of BA and CGA at the target site rely. The quantitative assessment of the combined pharmacological effect between the two agents in pharyngeal tissue warrants further investigation. This work not only provides a potential novel nano-formulation of TCM for the treatment of AP, but also opens a new avenue for the development and utilization of formulations based on the self-assembly of natural active components.

Nevertheless, several limitations of the present study should be acknowledged. Although BA-CGA@PM was included in the physicochemical characterization (e.g., XRD analysis), it was not incorporated into the biological evaluations. In addition, no formal pharmacological synergy analyses (e.g., combination index or isobolographic analysis) were performed. Therefore, the respective contributions of supramolecular self-assembly and simple compound combination to the enhanced biological effects could not be completely distinguished. Furthermore, an in vitro pharmacological positive control was not employed, and the chemically induced AP model does not fully recapitulate pathogen-specific acute pharyngitis. Although the involvement of the TLR4/MyD88/NF-*κ*B pathway was supported by WB analysis, the precise molecular targets and signaling mechanisms underlying the anti-inflammatory effects of BA-CGA@NPs remain to be further elucidated. Future studies incorporating appropriate biological control groups, mechanistic validation, formal synergy analyses, and infectious disease models are warranted to strengthen the translational relevance of this nano-formulation.

## Figures and Tables

**Table 1:** Pharmacokinetic parameters of BA before and after self-assembly ($\bar{x}$ ± SD, *n* = 6).

Parameter	Unit	BA	BA-CGA@NPs
*AUC*_(0–t)_	mg·h/L	74.37 ± 1.11	91.25 ± 4.20 **
*AUC*_(0–∞)_	mg·h/L	80.98 ± 1.12	92.26 ± 3.32 **
*MRT*_(0–t)_	h	9.52 ± 0.14	7.35 ± 0.08 **
*C*_max_	mg/L	8.16 ± 0.36	12.45 ± 0.96 **
*t*_1/2_	h	5.46 ± 0.06	2.88 ± 0.73 **
*T*_max_	h	6.67 ± 1.15	7.33 ± 1.15

Note: ** p < 0.01 vs. the BA group.

## Data Availability

The original contributions presented in this study are included in the article. Further inquiries can be directed to the corresponding authors.

## Supplementary Materials

The following supporting information can be downloaded at https://www.mdpi.com/article/10.3390/biomedicines14081724/s1, Figure S1: The discovery of self-assembly phenomenon in Pudilan; Figure S2: HPLC chromatograms (at 280 nm) of the test solution, the mixed standard reference solution and the single standard solution; Table S1: Linear regression equations, linear ranges, and correlation coefficients for CGA and BA; Table S2: Sample content determination of CGA and BA; Table S3: Standard curve parameters for BA and CGA; Table S4: Precision and accuracy analysis of plasma samples; Table S5: Recovery rate and matrix effect analysis; Table S6: Stability analysis of active pharmaceutical ingredients.

## Abbreviations

Baicalin, BA; Chlorogenic acid, CGA; Baicalin-chlorogenic acid nanoparticles, BA-CGA@NPs; Acute pharyngitis, AP; Physical mixture of BA and CGA, BA-CGA@PM; Hematoxylin–eosin staining, H&E; Sodium dodecyl sulfate-polyacrylamide gel electrophoresis, SDS-PAGE; Molecular dynamics simulation, MDS; van der Waals, vdW; Toll-like receptor 4, TLR4; Nuclear factor-*κ*B, NF-*κ*B; Inhibitor of NF-*κ*B, I*κ*B; Phosphorylated NF-*κ*B, p-NF-*κ*B; Phosphorylated I*κ*B*α*, p-I*κ*B*α*; Critical micelle concentration, CMC; Dynamic light scattering, DLS; Transmission electron microscopy, TEM; X-ray diffraction, XRD; Thermogravimetric analysis, TGA; Differential scanning calorimetry, DSC; Fourier-transform infrared, FT-IR; Lipopolysaccharide, LPS; Nitric oxide, NO; Reactive oxygen species, ROS; Peak plasma concentration, *C*_max_; Area under the concentration–time curve, *AUC*; Half-life period, *t*_1/2_; Mean residence time, MRT; Time to reach *C_max_*, *T*_max_; Western blot, WB.
