# Supplementary material for: Baicalin-Chlorogenic Acid Self-Assembled Nanoparticles: A Carrier-Free Nano-Formulation for the Treatment of Acute Pharyngitis
Source: Biomedicines. 2026 Jul 31;14(8):1724. doi: 10.3390/biomedicines14081724 (PMC13509274; doi:10.3390/biomedicines14081724)
Supplement: Supplementary file 1 [file biomedicines-14-01724-s001.zip › biomedicines-4374521-Supplementary material.pdf]

1. The discovery of self-assembly phenomenon in Pudilan

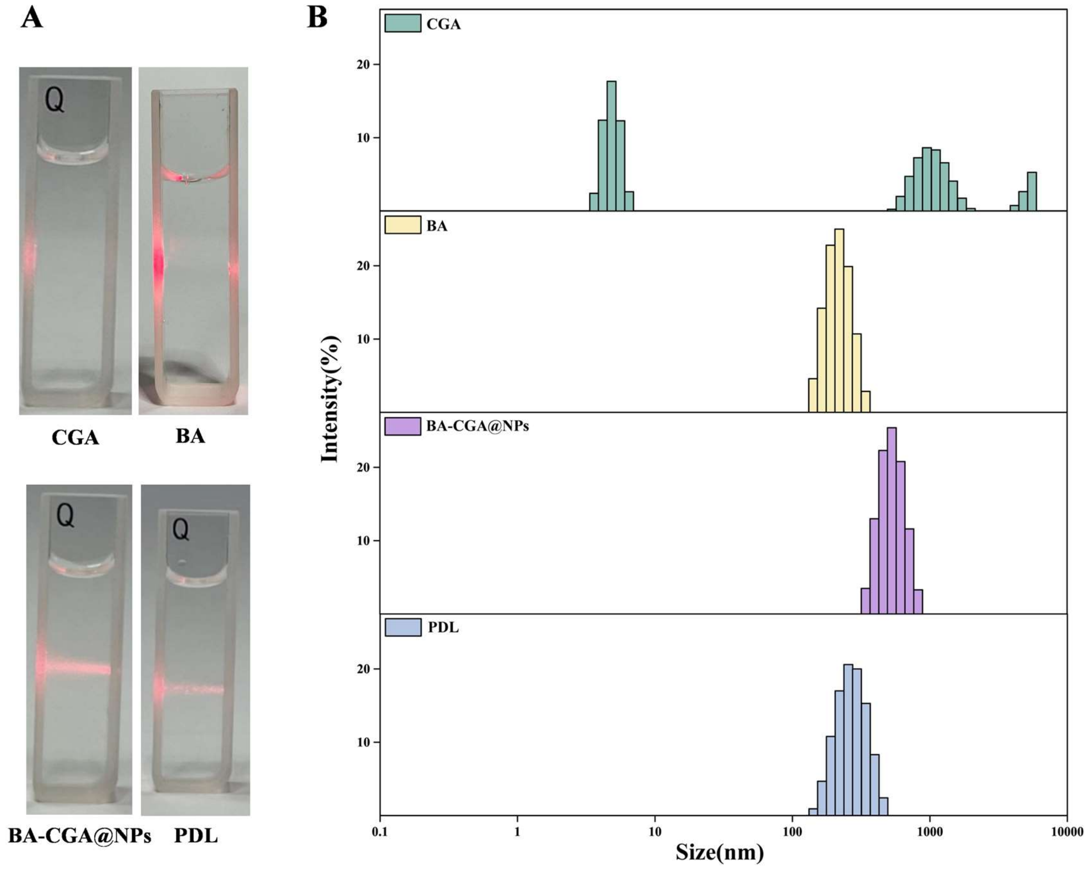

Fig. S1. The discovery of self-assembly phenomenon in Pudilan. (A)Tyndall effect characterization; (B) Particle size distribution

2. Determination of the contents of BA and CGA in BA-CGA@NPs

2.1. Method

2.1.1. Chromatographic Conditions

Chromatographic separation was performed on an Agilent 5 TC-C18 column (4.6 mm × 250 mm, 5 μm) maintained at 30 °C. The mobile phase consisted of 0.1% formic acid in water (A) and methanol (B). The gradient elution program was as follows: 0–1 min, 95% A; 1–5 min, 95% A → 48% A; 5–24 min, 48% A → 5% A; 24–29 min,

5% A → 95% A; 29–30 min, 95% A. The flow rate was 1 mL/min, the detection wavelength was set at 280 nm, and the injection volume was 10 μL.

2.1.2. Preparation of Mixed Reference Standard Solutions

BA (5 mg) and CGA (10 mg) were accurately weighed into a 10 mL volumetric flask, dissolved in methanol, and diluted to volume to obtain a mixed reference standard stock solution (BA 0.5 mg/mL, CGA 1 mg/mL). A 1.00 mL aliquot of this stock solution was serially diluted two-fold with methanol to yield mixed standard solutions of appropriate concentrations. All solutions were filtered through a 0.22 μm microporous membrane prior to use. Six calibration standards were prepared in this manner and stored for subsequent analysis.

2.1.3. Preparation of Test Solutions

An appropriate amount of BA-CGA@NPs was dissolved in methanol and filtered through a 0.22 μm microporous membrane to obtain the test solution stock.

2.1.4. Linearity Assessment

The six calibration standards (10 μL each) were injected under the chromatographic conditions described above, and the peak areas of the respective analytes were recorded. Linear regression was performed by plotting peak area (y) against mass concentration (x) to generate the calibration curves.

2.1.5. Precision Test

The mixed reference standard solution containing baicalin at 0.25 mg/mL and chlorogenic acid at 0.5 mg/mL was injected six consecutive times under the same chromatographic conditions. The peak areas were recorded, and the relative standard

deviation (RSD) was calculated to evaluate instrument precision.

2.1.6. Repeatability Test

Six replicate test solutions were prepared in parallel and analyzed under the above conditions. The peak areas were recorded, and the RSD was calculated.

2.1.7. Stability Test

A single test solution was injected after standing at room temperature for 0, 4, 8, 12, and 24 h under the same chromatographic conditions. The peak areas were recorded, and the RSD was calculated to assess sample stability.

2.2. Results and Discussion

2.2.1. Linearity Assessment

Appropriate volumes of the mixed standard stock solution were serially diluted two-fold with methanol to obtain a series of mixed calibration standard solutions. These solutions were injected under the chromatographic conditions described in Section 2.1.1, and the corresponding chromatographic data were acquired. Linear regression equations were constructed for each analyte by plotting the peak area (y) against the nominal concentration (x). The results are summarized in **Table S1**. Both analytes exhibited good linearity within the tested concentration ranges—0.03125–1 mg/mL for BA and 0.015625–0.5 mg/mL for CGA—with correlation coefficients (R^2) exceeding 0.9990.

Table S1 Linear regression equations, linear ranges, and correlation coefficients for CGA and BA.

Component	Concentration range (μg/mL)	Standard curve	R^2
CGA	31.25~1000.00	$y=1.47\times10^7x+2.31\times10^5$	0.9991
BA	15.63~500.00	$y=3.63\times10^7x+2.16\times10^5$	0.9993

2.2.2. Precision

The mixed reference standard solution was injected six consecutive times under the chromatographic conditions specified in Section 2.1.1. The peak areas of CGA and BA were recorded, and the relative standard deviations (RSD) were calculated. The RSD values for CGA and BA were 0.34% and 0.32%, respectively, both below 5%, indicating satisfactory instrument precision.

2.2.3. Repeatability

Six replicate test solutions were prepared in parallel according to the method described in "Preparation of Test Solutions" and analyzed under the chromatographic conditions given in **Section 2.1.1**. The contents of the two analytes were determined, and the RSD values were calculated. The RSD values for the content of CGA and BA were 1.60% and 1.11%, respectively, both below 5%, confirming good repeatability of the method.

2.2.4. Stability

A single test solution was injected after standing at room temperature for 0, 4, 8, 12, and 24 h under the chromatographic conditions described in Section 2.1. The peak areas were recorded, and the RSD was calculated. The RSD values were 1.32% for

CGA and 1.00% for BA, demonstrating that the test solution remained stable for 24 h at room temperature.

2.2.5. Sample Content Determination

The HPLC chromatograms of the test solution, reference standard solution, and mixed standard solution are shown in **Fig. S2**. The peak areas of each analyte were recorded and substituted into the respective linear regression equations to calculate their concentrations in the test solution **(Table S2)**. These values were subsequently converted to the molar ratio at which the two components self-assemble. The average contents of CGA and BA in the test solution were determined to be 0.183 mg/mL and 0.240 mg/mL, respectively, yielding a molar ratio of approximately 1:1.04. This experimentally determined ratio is in good agreement with the equimolar ratio employed in the molecular dynamics simulations (MDS).

Table S2 Sample content determination of CGA and BA.

	RT(min）	Area	Average area	Average content
CGA	8.489	2863028	2925619	0.18
	8.480	2917637		
	8.485	3006530		
	8.464	2906946		
	8.488	2927125		
	8.494	2932448		
BA	12.393	8905168	8939873	0.24
	12.379	8848100		
	12.388	9124795		
	12.365	8871230		
	12.391	8929119		
	12.392	8960826		

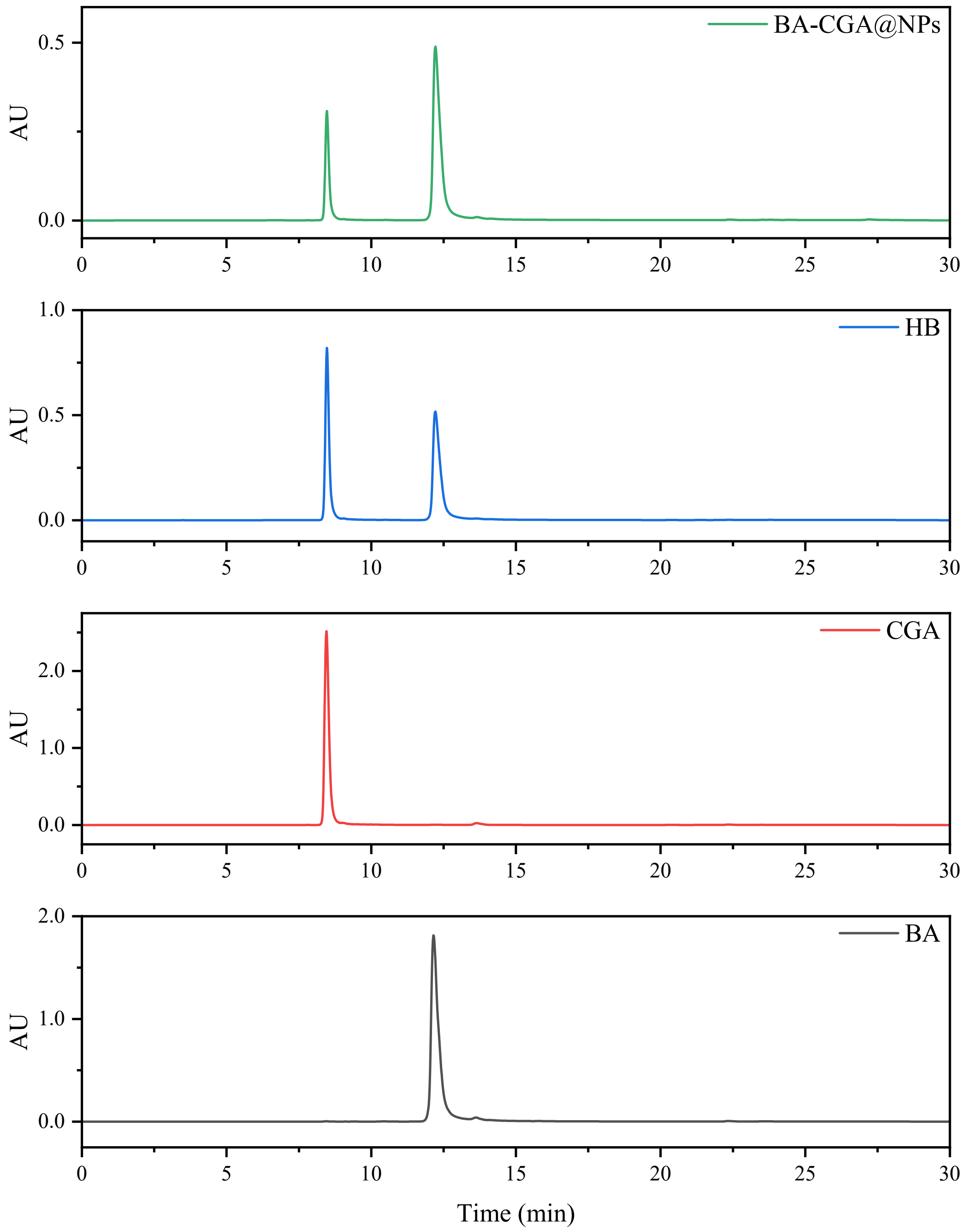

Fig. S2. HPLC chromatograms (at 280 nm) of the test solution, the mixed standard reference solution and the single standard solution

3. Methodological Investigation of the In Vivo Pharmacokinetics of BA-CGA@NPs

3.1. Method

3.1.1. Preparation of Reference Standard Solutions

CGA reference standard solution: 1 mg/mL (in methanol); BA reference standard solution: 1 mg/mL (in methanol); Mixed sample: CGA 0.5 mg/mL, BA 0.5 mg/mL (in methanol).

3.1.2. Plasma Sample Pretreatment Method

Plasma samples collected at various time points were thoroughly mixed. A 100 μL aliquot was precisely transferred to a 1.5 mL EP tube. Then, 30 μL of puerarin (internal standard, 1 μg/mL) and 750 μL of methanol were added for protein precipitation. The mixture was vortexed for 1 min and centrifuged at 13,000 rpm for 10 min. The supernatant was collected, dried under a gentle stream of nitrogen at 37°C, and the residue was reconstituted in 150 μL of methanol. After vortex mixing for 1 min, the solution was centrifuged again at 13,000 rpm for 10 min. The resulting supernatant was transferred into an insert vial for subsequent analysis.

3.1.3. Specificity Assessment

A 100 μL aliquot of blank rat plasma was mixed with 50 μL of methanol and then processed according to the method described in Section 2.1.2 to obtain the chromatogram of the blank sample. Subsequently, 100 μL of the mixed reference standard solution and 30 μL of the internal standard solution were added to blank plasma and treated following the same procedure to obtain the corresponding

chromatogram. Finally, plasma samples collected at 0.5 h from the BA-CGA@NPs group were injected and analyzed to assess whether endogenous components interfere with the detection of the target analytes.

3.1.4. Linearity and Limit of Quantification

The mixed sample was subjected to eight-step serial dilution. A 100 μL aliquot of blank plasma was spiked with 30 μL of each diluted level of the mixed sample and 30 μL of the internal standard solution, followed by sample processing according to the method described in **Section 2.1.2**. A standard curve was constructed by performing linear regression, where the peak area ratio of the analyte to the internal standard was plotted on the y-axis against the nominal concentration of each substance on the x-axis, yielding the corresponding regression equation.

3.1.5. Accuracy and Precision Assessment

Quality control (QC) samples: A 100 μL aliquot of blank rat plasma was precisely spiked with 50 μL of the mixed sample and 30 μL of the internal standard solution to prepare plasma QC samples at low, medium, and high concentration levels.

The accuracy and precision were evaluated using QC samples at these three concentration levels, with six replicate samples per concentration per batch. Precision was expressed as the relative standard deviation (RSD%). Accuracy was expressed as the relative error (RE%), calculated using the formula: RE% = [(theoretical concentration - measured concentration) / theoretical concentration] × 100%.

3.1.6. Matrix Effect and Sample Recovery Rates Assessment

A 100 μL aliquot of blank rat plasma was mixed with 500 μL of methanol and

vortexed for 1 min to completely precipitate proteins. After centrifugation at 13,000 rpm for 10 min, the supernatant was collected and spiked with working standard solutions at low, medium, and high concentration levels, along with the internal standard solution. The mixtures were then separately injected for analysis, and the obtained peak areas were recorded as A1.

Standard stock solutions at the low, medium, and high concentration levels were separately injected and analyzed, and the resulting peak areas were recorded as A2.

QC samples at the low, medium, and high concentration levels were separately injected and analyzed, and the resulting peak areas were recorded as A3.

Extraction recovery (%) = (A3 / A1) × 100%

Matrix effect (%) = (A1 / A2) × 100%

3.1.7. Stability Assessment

After processing, the QC samples were placed in an autosampler and injected at intervals over a 12-hour period to assess the stability of BA and CGA in the processed plasma samples under autosampler conditions (five replicates per concentration were analyzed). Using the same method, QC samples at high, medium, and low concentrations were prepared, subjected to three freeze-thaw cycles, and then processed for injection and concentration measurement to evaluate the stability of BA and CGA in plasma under repeated freeze-thaw conditions.

3.2. Results and Discussion

3.2.1. Investigation of Linear Relationships

The linear relationships for BA and CGA are shown in Table S3. The correlation

coefficients (R^2) were all ＞0.9990, indicating good linearity and meeting the requirements for quantification.

Table S3 Standard curve parameters for BA and CGA.

Component	Concentration range (μg/mL)	Standard curve	R^2
BA	0.61~49.96	y=0.554x-0.0536	0.9991
CGA	0.48~134.41	y=0.611x+0.0752	0.9991

3.2.2. Precision and Accuracy Assessment

The precision and accuracy for the target analytes, BA and CGA, in plasma samples are presented in **Table S4**. The RSD values were all within 5%, and the RE values were within 16%, indicating satisfactory instrument precision.

Table S4 Precision and accuracy analysis of plasma samples($\bar{x}$±SD，n=6).

Component	Concentration level	QC sample concentration (μg/mL)	Measured concentration (μg/mL)	RSD (%)	RE (%)
BA	Low	0.78	0.66±0.02	2.37%	15.22%
	Medium	3.12	3.30±0.11	3.19%	-5.57%
	High	12.49	10.85±0.18	1.70%	13.16%
CGA	Low	0.79	0.84±0.02	2.35%	-6.43%
	Medium	3.15	3.59±0.09	2.59%	-13.88%
	High	12.60	12.84±0.16	1.28%	-1.89%

3.2.3. Assessment of Recovery Rate and Matrix Effect

As shown in **Table S5**, the matrix effect for BA ranged from 66.51% to 106.14%, with RSD values within 5%, and its recovery rate ranged from 85.68% to 127.58%, with RSD values within 5%. For CGA, the matrix effect ranged from 81.53% to

139.89%, with RSD values within 7%, and its recovery rate ranged from 73.79% to 131.77%, with RSD values within 8%. These results indicate that the matrix effect had a limited impact on the target analytes, the recovery rates were acceptable, and the method generally meets the requirements for biological sample analysis.

Table S5 Recovery rate and matrix effect analysis ($\bar{x}$±SD，n=6).

Component	Concentration level	QC sample concentration (μg/mL)	Matrix Effect (%)	RSD (%)	Recovery Rate (%)	RSD (%)
	Low	0.78	66.51±2.40	3.61	127.58±4.66	3.65
BA	Medium	3.12	106.14±2.80	2.64	99.51±2.60	2.61
	High	12.49	101.38±1.78	1.75	85.68±1.50	1.75
	Low	0.79	81.53±3.03	3.72	131.77±4.80	3.64
CGA	Medium	3.15	105.95±0.80	0.76	108.40±0.82	0.76
	High	12.60	139.89±9.59	6.93	73.79±5.32	7.22

3.2.4. Stability Assessment

As shown in **Table S6**, the RE values for BA and CGA remained within 15%, and the RSD values were within 10% after 12 h of storage at room temperature and following repeated freeze-thaw cycles. These results indicate that the samples remained relatively stable under both room-temperature storage and repeated freeze-thaw conditions, meeting the requirements for routine analysis.

Table S6 Stability analysis of active pharmaceutical ingredients($\bar{x}$±SD，n=6).

Component	Concentration level	QC sample concentration (μg/mL)	Processed sample concentration (μg/mL)	RSD (%)	RE (%)
BA	Low	0.78	0.67±0.01	1.90	14.20%
	Medium	3.12	3.20±0.12	3.62	-2.45%
	High	12.49	10.71±0.19	1.76	14.28%
CGA	Low	0.79	0.77±0.01	1.09	2.22%
	Medium	3.15	3.39±0.30	8.90	-7.62%
	High	12.60	12.58±0.10	0.80	0.16%
